# The Major Floral Promoter NtFT5 in Tobacco (*Nicotiana tabacum*) Is a Promising Target for Crop Improvement

**DOI:** 10.3389/fpls.2019.01666

**Published:** 2020-01-10

**Authors:** Florentin J. Schmidt, Marius M. Zimmermann, David R. Wiedmann, Sophie Lichtenauer, Lena Grundmann, Jost Muth, Richard M. Twyman, Dirk Prüfer, Gundula A. Noll

**Affiliations:** ^1^ Institute of Plant Biology and Biotechnology, University of Münster, Münster, Germany; ^2^ Department of Functional and Applied Genomics, Fraunhofer Institute for Molecular Biology and Applied Ecology IME, Münster, Germany; ^3^ Department of Functional and Applied Genomics, Fraunhofer Institute for Molecular Biology and Applied Ecology IME, Aachen, Germany; ^4^ TRM Ltd, Scarborough, United Kingdom

**Keywords:** flowering locus T (FT), SR1, floral activator, long-day flowering, PEBP family, CRISPR/Cas9, loss-of-function-mutation, phloem companion cell-specific expression

## Abstract

The *FLOWERING LOCUS T* (*FT*)-like gene family encodes key regulators of flower induction that affect the timing of reproduction in many angiosperm species. Agricultural research has therefore focused on such genes to improve the success of breeding programs and enhance agronomic traits. We recently identified a novel *FT*-like gene (*NtFT5*) that encodes a day-neutral floral activator in the model tobacco crop *Nicotiana tabacum*. However, further characterization is necessary to determine its value as a target for breeding programs. We therefore investigated the function of NtFT5 by expression analysis and mutagenesis. Expression analysis revealed that *NtFT5* is transcribed in phloem companion cells, as is typical for *FT*-like genes. However, high levels of *NtFT5* mRNA accumulated not only in the leaves but also in the stem. Loss-of-function mutants (generated using CRISPR/Cas9) were unable to switch to reproductive growth under long-day conditions, indicating that NtFT5 is an indispensable major floral activator during long-days. Backcrossing was achieved by grafting the mutant scions onto wild-type rootstock, allowing the restoration of flowering and pollination by a wild-type donor. The resulting heterozygous *Ntft5^–^*/*NtFT5^+^* plants flowered with a mean delay of only ~2 days, demonstrating that one functional allele is sufficient for near-normal reproductive timing. However, this minor extension of the vegetative growth phase also conferred beneficial agronomic traits, including a >10% increase in vegetative leaf biomass on the main shoot and the production of more seeds. The agronomic benefits of the heterozygous plants persisted under various abiotic stress conditions, confirming that *NtFT5* is a promising target for crop improvement to address the effects of climate change.

## Introduction

Flowering is one of the major events in the life cycle of annual angiosperms, and the transition from vegetative to reproductive growth is an important agronomic trait in the context of plant breeding. The timing of flowering has a decisive influence on crop yield, including fruit ripening and the generation of biomass during vegetative growth. Recent studies have therefore focused on the molecular regulation of flowering time in various crop species (Jung and Müller, 2009; Blümel et al., 2015).

To optimize flowering under different environmental conditions, flowering plants coordinate the perception of endogenous and exogenous stimuli, including age, temperature, and day-length (Pajoro et al., 2014). The signals corresponding to these stimuli are integrated by the products of key genes such as *FLOWERING LOCUS T* (*FT*), a member of the phosphatidylethanolamine-binding protein (PEBP) family. These proteins are widely conserved regulators of developmental processes in plants and often function as activators of floral transition (Kardailsky et al., 1999; Kobayashi et al., 1999; Chardon and Damerval, 2005; Karlgren et al., 2011; Wickland and Hanzawa, 2015). The main regulator of the photoperiodic flowering pathway is the B-box zinc-finger transcription factor CONSTANS (CO), homologs of which are found in several important crop species, including potato, rice, and soybean (Putterill et al., 1995; Yano et al., 2000; Robson et al., 2001; González-Schain et al., 2012; Fan et al., 2014). In the model plant *Arabidopsis thaliana*, CO is stabilized by light in the afternoon solely under long-day (LD) conditions, causing the protein to accumulate and in turn induce the transcription of *FT* in the phloem companion cells of leaves (Samach et al., 2000; Suárez-López et al., 2001; Takada and Goto, 2003; An et al., 2004; Valverde et al., 2004; Wigge et al., 2005). As a result, the FT protein is exported into the phloem sieve elements and is transported to the shoot apical meristem (SAM), where it interacts with the bZIP transcription factor FD. The FT-FD complex activates the expression of floral meristem identity genes such as *APETALA1* (*AP1*), ultimately leading to the development of flowers (Abe et al., 2005; Wigge et al., 2005; Corbesier et al., 2007; Mathieu et al., 2007).

*FT*-like genes have been identified in several higher plant species, including important crops such as rice, sugar beet, potato, and tobacco (Kojima et al., 2002; Pin et al., 2010; Navarro et al., 2011; Harig et al., 2012; Beinecke et al., 2018). However, the influence of *FT*-like genes in these plants is not always limited to LD conditions. In rice, the *FT* ortholog *HEADING DATE 3A* (*HD3A*) specifically promotes flowering under short-day (SD) conditions. At the same time, the closely-related gene *RICE FLOWERING LOCUS T 1* (*RFT 1*) functions as a floral activator under both LD and SD conditions (Kojima et al., 2002; Komiya et al., 2008; Komiya et al., 2009). *FT* genes can also control developmental processes other than the floral transition. In potato, for example, *SELF PRUNING 6A* (*StSP6A*) and *StSP5G* regulate SD-specific tuberization in *S. tuberosum* ssp. *andigenum* genotypes, whereas *StSP3D* initiates day-neutral flowering (Navarro et al., 2011; Abelenda et al., 2016).

In some angiosperms, including sugar beet and tobacco, amino acid substitutions at key positions have converted certain FT homologs into floral repressors. Despite their sequence similarity to activator FTs, these proteins are functionally similar to another PEBP subfamily known as TERMINAL FLOWER 1 (TFL1)-like, whose members usually act as major repressors of flower induction (Ratcliffe et al., 1999; Pin et al., 2010; Harig et al., 2012; Ho and Weigel, 2014; Wickland and Hanzawa, 2015). At least in tobacco, antagonistic FTs may compete to bind conserved FD homologs at the protein level thereby fine-tune the timing of flowering (Beinecke et al., 2018).

In the day-neutral tobacco species *Nicotiana tabacum*, five *FT*-like genes (*NtFT1–NtFT5*) have been characterized thus far, three of which (*NtFT1*–*NtFT3*) encode floral repressors, whereas *NtFT4* and *NtFT5* encode floral activators (Harig et al., 2012; Beinecke et al., 2018; Wang et al., 2018). In *N. tabacum* cv. SR1, the NtFT1–NtFT4 proteins accumulate predominantly under SD conditions and may thus regulate flowering during short days. In contrast, *NtFT5* is expressed regardless of the day length, indicating a regulatory function under both LD and SD conditions. NtFT5 is therefore the only *N. tabacum* FT homolog identified thus far that induces flower development under agriculturally-relevant LD conditions (Beinecke et al., 2018).

Here we investigated the role of NtFT5, focusing on its importance for the regulation of flowering time under LD conditions. We carried out a comprehensive analysis of gene expression at the tissue level by real-time quantitative PCR (qPCR) and by using the *NtFT5* upstream promoter to drive the expression of the reporter genes *uidA* and *GFP_ER_*, encoding β-glucuronidase (GUS) and endoplasmic reticulum-localized green fluorescent protein (GFP_ER_), respectively. We also used the CRISPR/Cas9 genome-editing system to create loss-of-function mutants of *NtFT5* and compared the agronomic phenotypes of *NtFT5^+^* wild-type plants and plants heterozygous for the null *Ntft5^–^* allele under normal conditions and various forms of stress to evaluate the potential of this gene as a breeding target for the improvement of crops.

## Materials and Methods

### Plant Materials and Growth Conditions

The tobacco species *N. tabacum* cv. SR1 (IPK, Gatersleben, Germany) was used as a model in this study. For the analysis of *NtFT5* expression, wild-type tobacco seeds were sown and the plants were cultivated in soil under LD conditions in the greenhouse (16-h photoperiod, artificial light switched on if natural light fell below 700 μmol m^−2^ s^−1^, 22–25°C under light, 19–25°C in the dark), or under SD conditions in phytochambers (8-h photoperiod, 200 μmol m^−2^ s^−1^, 25–27°C under light, 20°C in the dark). Apical, medial, and basal leaves, as well as stems, were separately harvested from vegetative plants with 7–9 leaves (week 4.5, LD) or 11–12 leaves (week 6, SD), and from plants with floral buds (week 6.5, LD or week 8, SD). Immediately at the beginning of the light period, three samples (biological replicates) were taken per tissue and time point, each comprising material pooled from three individual plants. The harvested plant material was snap-frozen and ground in liquid nitrogen, and stored at –80°C.

For *Agrobacterium*-mediated transformation, wild-type plants were germinated and grown under sterile conditions (LD, 16-h photoperiod, 23°C, 100 µmol m^−2^ s^−1^) on MS medium (Murashige and Skoog, 1962).

For promoter studies, T_1_ plants of independent lines carrying the P*_NtFT5_:uidA* or P*_NtFT5_:GFP_ER_* constructs were germinated in a sterile environment under LD conditions (as above) on MS medium containing 25 mg/L hygromycin B. Growing plants were transferred to pots containing soil and were cultivated under LD conditions in the greenhouse.

CRISPR/Cas9 *NtFT5* knockout plants were analyzed by cultivating the T_1_ and backcross generations 1 and 2 (BC_1_ and BC_2_) of one flowering T_0_ plant line, which carried loss-of-function *Ntft5^–^* alleles. The seeds were sown in soil and the plants were grown under LD conditions in the greenhouse. Seed production in the selected non-flowering homozygous *Ntft5^–^* T_1_ individual was induced by grafting it as a scion onto SR1 wild-type rootstock, replacing the upper part of the main shoot, which had already generated buds or flowers. The developing flowers of the scion were backcrossed, using a wild-type pollen donor. Selfing was prevented during backcrossing by removing the immature stamens.

For the phenotyping of homozygous *NtFT5^+^* and heterozygous *Ntft5^–^*/*NtFT5^+^* BC_2_ individuals under abiotic stress conditions, plants were sown and initially grown under normal LD conditions as described above. After 30 days, the plants were assigned to subgroups, shifted to the different abiotic stress conditions listed in **Table 1**, and cultivated until the flowering stage for phenotypic analysis.

**Table 1:** Summary of conditions used for phenotyping under the influence of abiotic stress. Plants were grown under normal LD conditions and stress treatment was initiated 30 days after seed sowing (DASS). Phenotyping was carried out when all plants grown under the same abiotic stress conditions had opened their first flowers.

Condition	Temperature [°C]	Watering	Time point of phenotyping [DASS]
Normal conditions (control)	Light 0–16 h 22–25°C, dark 16–0 h 19–25°C	Flooding of the desks for 10 min (three times daily 1, 5 and 9 h after beginning of the light period)	61
Salt		Normal conditions, the water was supplemented with 100 mM NaCl	63
Drought		2.9 L per plant over a period of 35 days, with 50–300 mL every 2–3 days depending on the size and wilting of the plants	72
Waterlogging		Constant water level of 7–8 cm on the desks	61
Heat	Light 0–6 h 30°C 6–12 h 40°C 12–16 h 30°C, dark 16–0 h 25°C	Normal conditions	63
Heat and drought		3.6 L per plant over a period of 47 days, with 50–300 mL every 2–3 days depending on the size and wilting of plants	84
Heat and waterlogging		Constant water level of 7–8 cm on the desks	68

### Phenotyping the BC_2_ Generation of *NtFT5* Knockout Plants

Under normal LD conditions (see above), flowering homozygous *NtFT5^+^* and heterozygous *Ntft5^–^*/*NtFT5^+^* BC_2_ individuals were compared in terms of height, number of leaves, and amount of vegetative biomass on the main shoot ~8 weeks after seed sowing (WASS), representing an early flowering stage immediately after all individuals had opened their first flowers. Days until flowering was defined as the period between seed sowing and the day the first flower opened. The height of the plants was determined by measuring the stem length (base of the pot to the main inflorescence). The vegetative biomass of the main shoot was measured by separately harvesting stem and leaves and weighing the fresh material. The dry weight of the leaves was measured after drying in a heating cabinet. The number of lateral shoots was determined ~13.5 WASS, when the plants had already generated ripened capsules. The capsules produced by the main and lateral shoots were harvested separately ~15 WASS to determine the total seed weight after drying. We also measured the thousand grain weight (TGW) using an Elmor C3 Seed Counter running Elmor C3 Counter v1.8.9.512 software (Elmor, Schwyz, Switzerland) in combination with a BJ100M micro scale (Precisa Gravimetrics, Dietikon, Switzerland). Duplicate TGW measurements were acquired and averaged, each comprising ~5,000 seeds.

Comparative phenotyping of flowering homozygous *NtFT5^+^* and heterozygous *Ntft5^–^*/*NtFT5^+^* BC_2_ individuals growing under various forms of abiotic stress was carried out at an early flowering stage when all plants growing under the same abiotic stress conditions produced their first open flowers (**Table 1**). The days until flowering, number of leaves, plant height, and the amount of vegetative fresh and dry leaf biomass on the main shoot were determined as described above. The results we previously observed during initial phenotyping under normal LD conditions (**Figures 5** and **6**) were confirmed and total percentages were even exceeded, which can most probably be attributed to slight variation in overall growth conditions due to cultivation in a different green house.

### Extraction of Nucleic Acids and cDNA Synthesis

Genomic DNA for PCR analysis was extracted from fresh leaf material ground in the corresponding lysis buffer using an MM400 bead mill (Retsch, Haan, Germany). To isolate the *NtFT5* genomic sequence, genomic DNA was purified using the NucleoSpin Plant II kit (Macherey-Nagel, Düren, Germany). To screen P*_NtFT5_*-reporter gene lines and to genotype the *NtFT5*-knockout plants (generations T_0_, T_1_ and BC_1_), genomic DNA was isolated as previously described (Edwards et al., 1991). The analysis of *NtFT5*-knockout BC_2_ plants required a 96-well plate format, so we used the Chemagic DNA Plant kit for extraction (PerkinElmer, Waltham, MA, USA) combined with an appropriate magnetic stand and a rotary shaker for resuspension of the beads.

For the analysis of *NtFT5* expression, plant tissue was ground in a mortar under liquid nitrogen and total RNA was isolated using the innuPREP Plant RNA kit (Analytik Jena, Jena, Germany). Residual genomic DNA was digested using the TURBO DNA-free kit (Thermo Fisher Scientific, Waltham, MA, USA). The RNA quality was determined using a NanoPhotometer UV/Vis spectrophotometer (Implen, Munich, Germany) and by agarose gel electrophoresis. The RNA (final concentration 50 ng/mL) was converted into cDNA using PrimeScript RT Master Mix (Perfect Real Time) (Takara Bio Europe, Saint-Germain-en-Laye, France).

### Isolation of the Genomic *NtFT5* Locus

The *NtFT5* genomic locus including ~2.74 kb of the promoter (P*_NtFT5_*) was amplified from *N. tabacum* cv. SR1 genomic DNA in five overlapping fragments. The parts were separately amplified by PCR using gene-specific primers (**Supplementary Table S1**). The primer sequences were designed *in silico* based on the *NtFT5* locus previously identified in the published *N. tabacum* cv. Basma Xanthi genome (Sierro et al., 2014; Beinecke et al., 2018). The resulting PCR products were adenylated using MangoTaq DNA polymerase (Bioline, London, UK), transferred to pCRII-TOPO using the TOPO TA Cloning kit (Thermo Fisher Scientific) and sequenced. The full-length genomic sequence of *NtFT5* was then assembled *in silico* using SeqManPro and SeqBuilder Pro in Lasergene v15 (DNASTAR, Madison, WI, USA). The same software was used to determine the gene structure by aligning the genomic clone with the previously-described coding sequence: GenBank KY306470.1 (Beinecke et al., 2018).

### Cloning the P*_Nt__FT5_*-Reporter Gene and CRISPR/Cas9 Constructs

For promoter studies, 2.57 kb of the P*_NtFT5_* sequence was fused to the reporter genes *uidA* and *GFP_ER_*. The genomic sequence upstream of *NtFT5* was amplified by PCR using Phusion High-Fidelity DNA Polymerase and specific primers containing appropriate restriction sites (**Supplementary Table S1**) for the entry vectors pBsGFP_ER_ (Noll et al., 2007) and pBsGUS. The latter was based on pBluescript II KS (+) (Stratagene Cloning Systems, La Jolla, CA, USA) and contained the *uidA* gene and the CaMV T_35S_ from pAM-CaMV35S-GUS (Fricke et al., 2013). The P*_NtFT5_* PCR products and the vectors were digested using KpnI/XhoI and P*_NtFT5_* was inserted upstream of each reporter gene. Finally, the P*_NtFT5_*:*uidA*/CaMV T*_35S_* and P*_NtFT5_*:*GFP_ER_*/CaMV T*_35S_* cassettes were cut out with KpnI/SacI and ligated to the appropriately linearized binary vector pBin19Hyg (Bevan, 1984), which contained *hpt* instead of *nptII* as the selectable marker gene and was kindly provided by Dr. Lena Grundmann (University of Münster, Münster, Germany).

For the targeted editing of *NtFT5*, the binary construct pDe-CAS9-*NtFT5*_ex__I-147.169__bp_ was generated as previously described (Fauser et al., 2014). The original vectors were kindly provided by Holger Puchta (Karlsruhe Institute of Technology, Karlsruhe, Germany). The *NtFT5*-specific protospacer, targeting a site in exon I, was derived *in silico* using the target online predictor CCTop (Stemmer et al., 2015; http://crispr.cos.uni-heidelberg.de) based on the genome sequence of *N. tabacum* cv. Basma Xanthi (Sierro et al., 2014). Potential protospacer off-targets were predicted by screening the tobacco genome with CCTop for related sites with NGG-type and NRG-type protospacer adjacent motifs (PAMs). Only sequences containing a maximum of two mismatches in the 12-bp core region of the protospacer and four mismatches in total were considered as potential off-targets (Stemmer et al., 2015). The protospacer was generated by annealing two single-stranded oligonucleotides (**Supplementary Table S1**) including 5′ overhangs compatible with BbsI-linearized entry vector pEn-Chimera (Fauser et al., 2014). The cassette containing the *NtFT5*-specific sgRNA was then transferred to the destination vector pDe-Cas9 (Fauser et al., 2014) using Gateway LR Clonase II Enzyme Mix (Thermo Fisher Scientific).

### *Agrobacterium*-Mediated Tobacco Transformation

Stable plant transformation was carried out using the leaf disc method (Horsch et al., 1985) with *Agrobacterium tumefaciens* strains LBA4404 (Hoekema et al., 1983) for P*_NtFT5_* reporter gene studies and EHA105 (Hood et al., 1993) for CRISPR/Cas9-mediated genome editing. The appropriate binary vectors were introduced into these strains by electroporation. For the selection of transgenic plants, MS medium was supplemented with 25 mg/L hygromycin B or 3 mg/L phosphinothricin, as appropriate. After callus regeneration and rooting in sterile culture medium, independent transgenic plant lines were cultivated in soil under LD conditions in the greenhouse as described above.

### Identification and Screening of Genome-Edited Transgenic Plants

After regeneration from callus, transgenic T_0_ plant lines were identified by PCR (MangoTaq DNA polymerase) using genomic DNA as the template and the primers listed in **Supplementary Table S1**. Plants of the *NtFT5* knockout generations T_0_ and T_1_ were screened to determine the presence of the *cas9* gene, with the *N. tabacum glyceraldehyde-3-phosphate dehydrogenase* (*NtGAPDH*) gene used as a control.

For the analysis of genome editing, *NtFT5* exon I was amplified from genomic DNA using MyTaq DNA polymerase (Bioline) and the primers listed in **Supplementary Table S1**. For chimeric T_0_ plants, the amplicons were transferred to pCRII-TOPO (TOPO TA Cloning kit) and sequenced. Starting with T_1_ individuals lacking *cas9*, plants were genotyped by direct sequencing of the purified amplicons. Individuals of the T_1_ and BC_1_ generation were also genotyped by fragment length analysis of the *NtFT5* genomic sequence. The latter was carried out with shorter fragments of exon I, which were amplified using primers (see **Supplementary Table S1**) carrying the fluorescent dye 6-carboxyfluorescein (6-FAM). To identify mutated allele variants with indels, the amplicon length of the native *NtFT5* allele was simultaneously determined and used as a reference. The PCR samples were mixed with the GeneScan 600 LIZ dye Size Standard v2.0 (Thermo Fisher Scientific) and analyzed in an ABI 3730 Genetic Analyzer (Applied Biosystems, Waltham, MA, USA). The data were evaluated using GeneMarker v2.6.4 (SoftGenetics, State College, PA, USA).

To rule out off-target effects on related *FT*-like genes, the potential off-targets were genotyped in the selected T_1_ generation plant (#78) by the direct sequencing of purified amplicons using the primer combinations in **Supplementary Table S1**.

### Spatiotemporal Expression Analysis by qPCR

The spatiotemporal expression of *NtFT5* was analyzed by qPCR in a C1000 Touch Thermal Cycler using the CFX 96 Real-Time System (Bio-Rad Laboratories, Hercules, CA, USA) and KAPA SYBR FAST qPCR Master Mix (Merck, Darmstadt, Germany). For each reaction, 2.5 µl of cDNA (diluted 1:10; equivalent to ~12.5 ng) served as the template and the final concentration of each primer was 500 nM. The qPCR program comprised an initial denaturation step (95°C, 3 min), followed by 40 cycles of denaturation (95°C, 3 s) and annealing/extension for 30 s at primer-specific temperatures (**Supplementary Table S1**). Melting curve analysis (5 s, 58–95°C, ΔT = 0.5°C) was carried out to ensure amplicon specificity. The three biological replicates per tissue and harvesting time point were each analyzed in technical triplicates for each gene. The no-reverse-transcriptase (NRT) and no-template controls (NTC) were carried out in duplicates. Bio-Rad CFX Manager v3.1 (Bio-Rad Laboratories) was used for data analysis. The quantification cycle (Cq) values of the technical triplicates were averaged and used to determine the mean of the three biological replicates. The expression ratio of *NtFT5* was calculated as previously described (Livak and Schmittgen, 2001). The reference gene *N. tabacum elongation factor 1α* (*NtEF-1α*) was used for normalization (Schmidt and Delaney, 2010). Between-run variation was removed by factor correction (Ruijter et al., 2015).

### P*_Nt__FT5_*-Reporter Gene Studies

The P*_NtFT5_*:*GFP_ER_*/CaMV T*_35S_* T_1_ plants were analyzed by confocal laser scanning microscopy (CLSM) using a Leica TCS SP5 X microscope (Leica Microsystems, Wetzlar, Germany). The callose-specific dye aniline blue was used to visualize the sieve tube plates of the phloem by incubating longitudinal sections of the stem for up to 5 min in staining solution (0.1% (w/v) aniline blue in a 1:1 (v/v) mix of glycerol/deionized water) and the sections were washed in the same solution without dye. Fluorescence signals were measured at excitation and emission wavelengths of 488 and 500–600 nm (GFP_ER_), or 405 nm and 479–533 nm (aniline blue), respectively.

Promoter activity in P*_NtFT5_*:*uidA*/CaMV T*_35S_* T_1_ individuals was detected by the histochemical analysis of GUS activity. Several stem and leaf petiole sections, as well as leaf disks punched from laminae were infiltrated (1 min vacuum) with staining solution based on sodium phosphate buffer (57.6 mM Na_2_HPO_4_, 42.2 mM NaH_2_PO_4_, pH ~7.0) containing 5 mM K_3_Fe(CN_6_), 5 mM K_4_Fe(CN_6_) and 2.875 mM X-GlcA. The sections were incubated for up to 24 h at 37°C. Chlorophyll was extracted from the samples by incubation in methanol (37°C for up to 3 h). The samples were stored in deionized water at 4°C prior to imaging using a MZ 16 F stereomicroscope (Leica Microsystems).

### Accession Numbers

The genomic *NtFT5* sequence data are available in the GenBank data library under accession number MK910742. The GenBank accession numbers of the *NtFT1*–*NtFT13* coding sequences (Harig et al., 2012; Beinecke et al., 2018) used for CRISPR/Cas9 off-target analysis are listed in **Supplementary Table S5**.

## Results

### *NtFT5* is Expressed in Phloem Companion Cells in the Stem and Leaves

**Figure 1:**
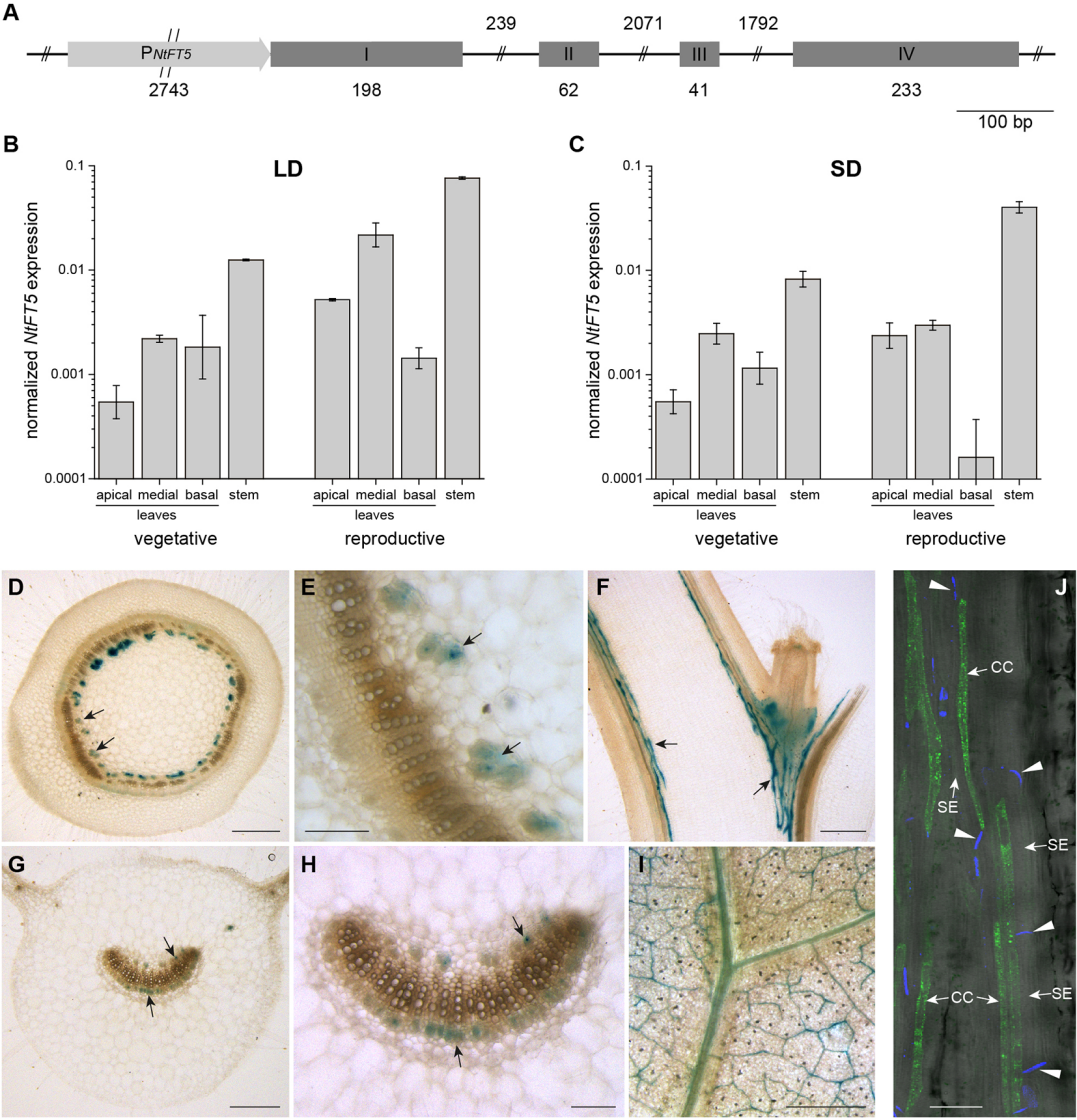
*NtFT5* gene structure, spatiotemporal expression, and cell-specific analysis of promoter activity in *N. tabacum* cv. SR1 plants. **(A)** Structure of the *NtFT5* gene including ~2.74 kb of the promoter. Exons are shown as boxes, introns as lines, and the promoter as an arrow. **(B, C)** Spatiotemporal expression of *NtFT5* in the leaves and stem of vegetative and reproductive wild-type plants (cv. SR1) analyzed by quantitative real-time PCR (qPCR) under long day (LD) **(B)** and short day (SD) **(C)** conditions. *NtFT5* expression was normalized to the reference gene *NtEF-1α*. Results are shown as mean values of three biological replicates (n = 3) ± standard error of the mean (SEM, error bars) based on log-transformed data, with the exception n = 2 for basal leaves of reproductive plants under SD conditions. **(D–I)** GUS activity in the stem and leaves of transgenic P*_NtFT5_*:*uidA* plants. Stained phloem tissue is representatively indicated with arrows. The panels show cross sections **(D, E)** and longitudinal sections **(F)** of the stem, as well as petiole cross sections **(G**–**H)** and laminal disks **(I)** of medial leaves. **(J)** GFP_ER_ fluorescence in transgenic P*_NtFT5_*:*GFP_ER_* plants analyzed by CLSM. Fluorescence is abundant in phloem companion cells (CC) adjacent to sieve elements (SE) in the petioles and stem (a representative longitudinal stem section is shown). The callose-containing sieve plates (indicated with arrow heads) are stained with aniline blue. **(D–J)** The representative tissue sections were prepared from T_1_ plants cultivated under LD conditions ~3 weeks (P*_NtFT5_*:*uidA*, L10) and ~5 weeks (P*_NtFT5_*:*GFP_ER_*_,_ L1) after transfer from sterile culture to the greenhouse. Scale bars: **(D, F, G, I)** 1 mm; **(E, H)** 250 µm; **(J)** 50 µm.

The gene structure of *NtFT5* was recently reported in *N. tabacum* cv. HongHuaDaJinYuan (Wang et al., 2018). To verify the sequence and investigate the regulatory function of NtFT5 in more detail, we isolated the *NtFT5* genomic locus from *N. tabacum* cv. SR1, including ~2.74 kb of the upstream promoter (**Figure 1A**). To achieve this, we designed primers based on the corresponding sequence, which we recently identified *in silico* in the *N. tabacum* genome (Beinecke et al., 2018). The exon structure of the *NtFT5* gene in cv. SR1 was identical to that previously described for cv. HongHuaDaJinYuan, comprising four exons (I–IV) although the intron sizes showed some minor differences (Wang et al., 2018). The sizes of exons II and III were typical of *FT*-like genes and are conserved in all other *NtFT* genes that have been analyzed thus far (Kobayashi et al., 1999; Harig et al., 2012).

We investigated the expression profile of *NtFT5* in SR1 plants by qPCR and promoter reporter gene analysis. Because the abundance of the *NtFT5* transcript is photoperiod-independent (Beinecke et al., 2018), we used qPCR to compare expression levels in the leaves and stem at two developmental stages under LD (**Figure 1B**) and SD (**Figure 1C**) conditions. We found that *NtFT5* was not only expressed in the apical, medial, and basal leaves of SR1 plants, but the transcript was also remarkably abundant in the stem. We also observed a slight tendency towards higher expression levels in the reproductive plants, which was detectable under both LD and SD conditions predominantly in the apical leaves and stem.

The cell-type specific expression of *NtFT5* was investigated by generating two expression cassettes containing 2.57 kb of the *NtFT5* promoter sequence (P*_NtFT5_*) fused either to the *uidA* reporter gene encoding GUS, or to *GFP_ER_*. Because our key objective was to characterize *NtFT5* under agriculturally-relevant LD conditions, we chose these conditions for the analysis of promoter activity in transgenic plants. We initially stained for GUS in the stem (**Figures 1D–F**) and leaves (**Figures 1G–I**), which revealed promoter activity in the vascular bundles, specifically the phloem, in both tissues of three transgenic P_NtFT5_:*uidA* lines (P_NtFT5_:*uidA* L10 is shown as a representative example in **Figure 1**). In medial leaves, P*_NtFT5_* activity was not restricted to the petiole (**Figures 1G, H**), but was also detected in the smaller vascular bundles of the lamina (**Figure 1I**). Moreover, fluorescence microscopy of longitudinal leaf petiole and stem sections prepared from P*_NtFT5_*:*GFP_ER_* plants indicated that the observed phloem-specific histochemical staining could be traced to the expression of *NtFT5* in phloem companion cells (a representative stem section of P*_NtFT5_*:*GFP_ER_* L1 is shown in **Figure 1J**).

### Analysis of *NtFT5* Mutations Generated Using the CRISPR/Cas9 System

**Figure 2:**
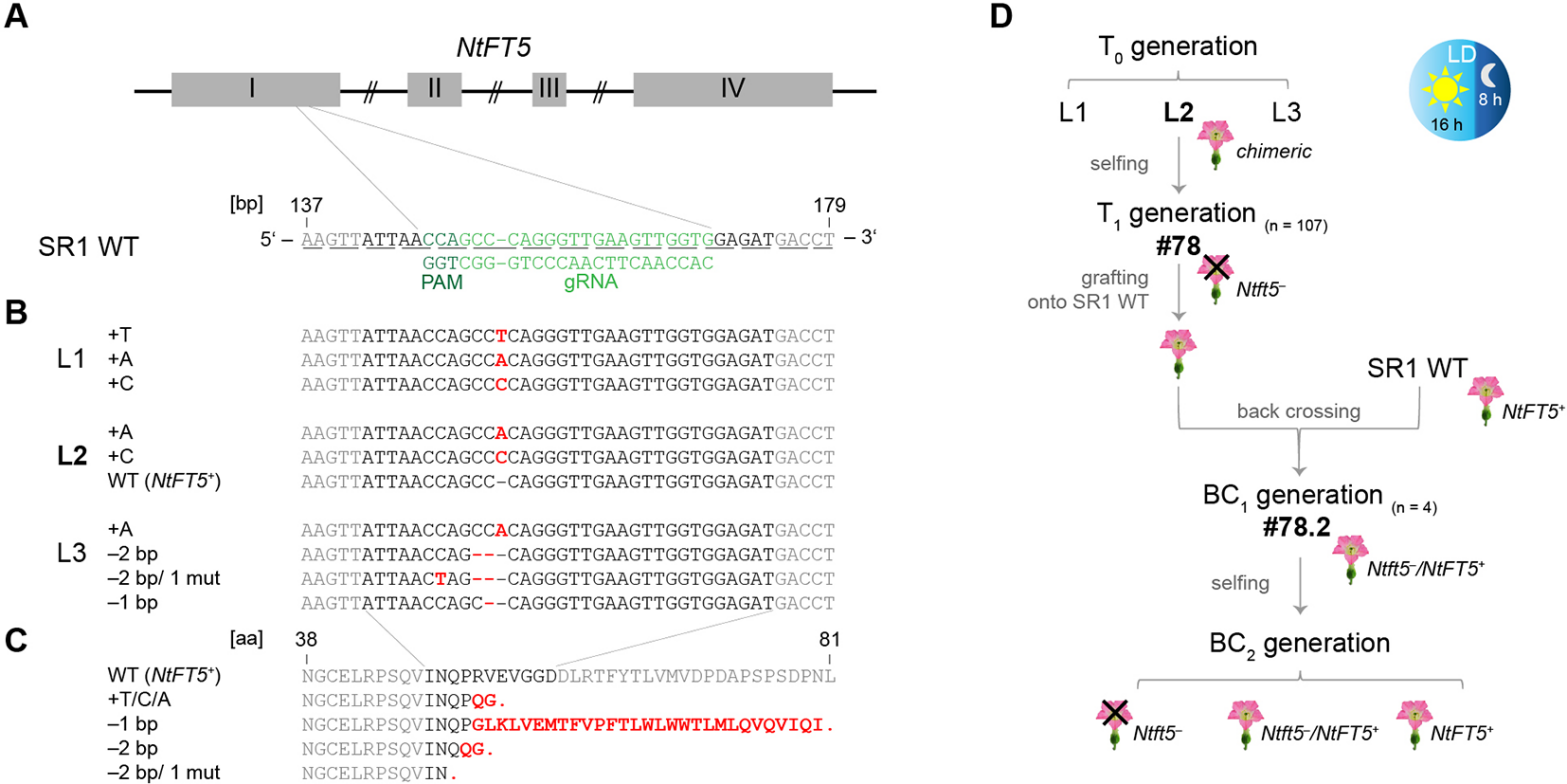
CRISPR/Cas9 genome editing of *NtFT5* in *N. tabacum* cv. SR1 and identification of induced mutations in T_0_ plants. **(A)** Location of the *NtFT5*-specific protospacer and the protospacer adjacent motif (PAM) on the antisense DNA strand in the 147–169 bp region of exon I. Exons are shown as boxes, introns as lines. The underlines indicate triplets corresponding to amino acids. **(B)** PCR-based screening of three independent transgenic T_0_ lines (L1–L3) for mutated allelic variants of *NtFT5*. *NtFT5* (exon I) was amplified using the primer combination listed in **Supplemental Table S1**, single amplicons were transferred to pCRII-TOPO and sequenced (n = 15 per line). Point mutations (mut), insertions and deletions are highlighted in red letters. **(C)** Sequence alignment of native NtFT5 with the protein variants encoded by the identified mutated alleles. Red letters indicate amino acid substitutions and red stops indicate premature termination. **(D)** Experimental workflow of *NtFT5* knockout analysis under LD conditions. Flowering T_0_ plant L2 was chosen for detailed characterization. In the T_1_ generation (n = 107) all characterized individuals lacking the *cas9* gene (n = 7) were non-flowering and showed a nullizygous *Ntft5^–^* genotype. T_1_ plant #78 was representatively selected for backcrossing, and flowering was induced by grafting onto wild-type (*NtFT5^+^*) rootstock. After pollination using a wild-type donor, heterozygous *Ntft5^–^/NtFT5^+^* plants of the first backcross generation (BC_1_, n = 4) flowered and one of these (#2) was randomly self-fertilized. Detailed phenotyping of non-flowering nullizygous *Ntft5^–^* plants and flowering *NtFT5^+^* and *Ntft5^–^/NtFT5^+^*plants was finally carried out in the second backcross generation (BC_2_).

The *NtFT5* gene was targeted for mutagenesis using CRISPR/Cas9 technology in SR1 plants to evaluate its potential as breeding target for crop improvement. To induce proximal frameshift mutations, we identified several potential protospacers specifically targeting sites in the first two exons of *NtFT5*. The genomic sequence of *N. tabacum* cv. Basma Xanthi (Sierro et al., 2014) was used as a reference and was screened for potential off-target sites, especially in the *NtFT* coding regions. Based on this *in silico* analysis, we chose one of the protospacers located on the antisense DNA strand of *NtFT5*, and targeted a site between positions 147 and 169 in the middle of exon I (**Figure 2A**). The closely-related *NtFT7* gene is not found in SR1 plants (Beinecke et al., 2018), hence the corresponding off-target site for our protospacer is absent in this cultivar. Furthermore, the protospacer showed at least two mismatches compared to any other potential exonic off-target, including the coding sequences of all other *NtFT* genes (*NtFT1–NtFT13*) identified thus far (Harig et al., 2012; Beinecke et al., 2018), suggesting that off-target mutations were unlikely. We generated the corresponding pDe-CAS9-*NtFT5*_ex__I-147.169__bp_ construct containing the chosen protospacer as part of the sgRNA.

Stable transformation of tobacco plants using the construct described above yielded several independent transgenic T_0_ plant lines, all of which carried an integrated copy of the *cas9* transgene as part of the introduced T-DNA, providing the basis for *NtFT5* editing (**Supplementary Figure S1A**). Three of these lines (L1–L3) were analyzed to determine their genotype. Given that these plants were likely to be chimeras due to ongoing Cas9 activity, we sequenced various single PCR amplicons of the genomic *NtFT5* locus, focusing on the corresponding part in exon I. Accordingly, mutated allele variants of *NtFT5* were identified in all three lines, indicating that genome editing was successful. The induced mutations were indels of –2, –1 and +1 bp at the chosen target site. L1 and L3 carried multiple mutant alleles whereas only L2 also carried the wild-type *NtFT5* allele (**Figure 2B**). Alignment of the native NtFT5 protein and its new allelic variants showed that the induced mutations led to early stop codons resulting in truncated proteins of different lengths (**Figure 2C**). However, the identification of more than two different allele variants per line confirmed the chimeric nature of the T_0_ plants and we therefore selected only *cas9*-free *NtFT5* knockout individuals for phenotypic analysis in subsequent plant generations. Accordingly, the following experiments (the workflow is shown in **Figure 2D**) were performed with the offspring of one *NtFT5* knockout T_0_ plant (L2) since L1 and L3 were non-flowering. L2 still flowered, probably reflecting the presence of the wild-type *NtFT5* allele, but the production of fertile flowers was delayed under LD conditions.

### Homozygous *NtFT5* Knockout Prevents LD Flowering in SR1 Plants

**Figure 3:**
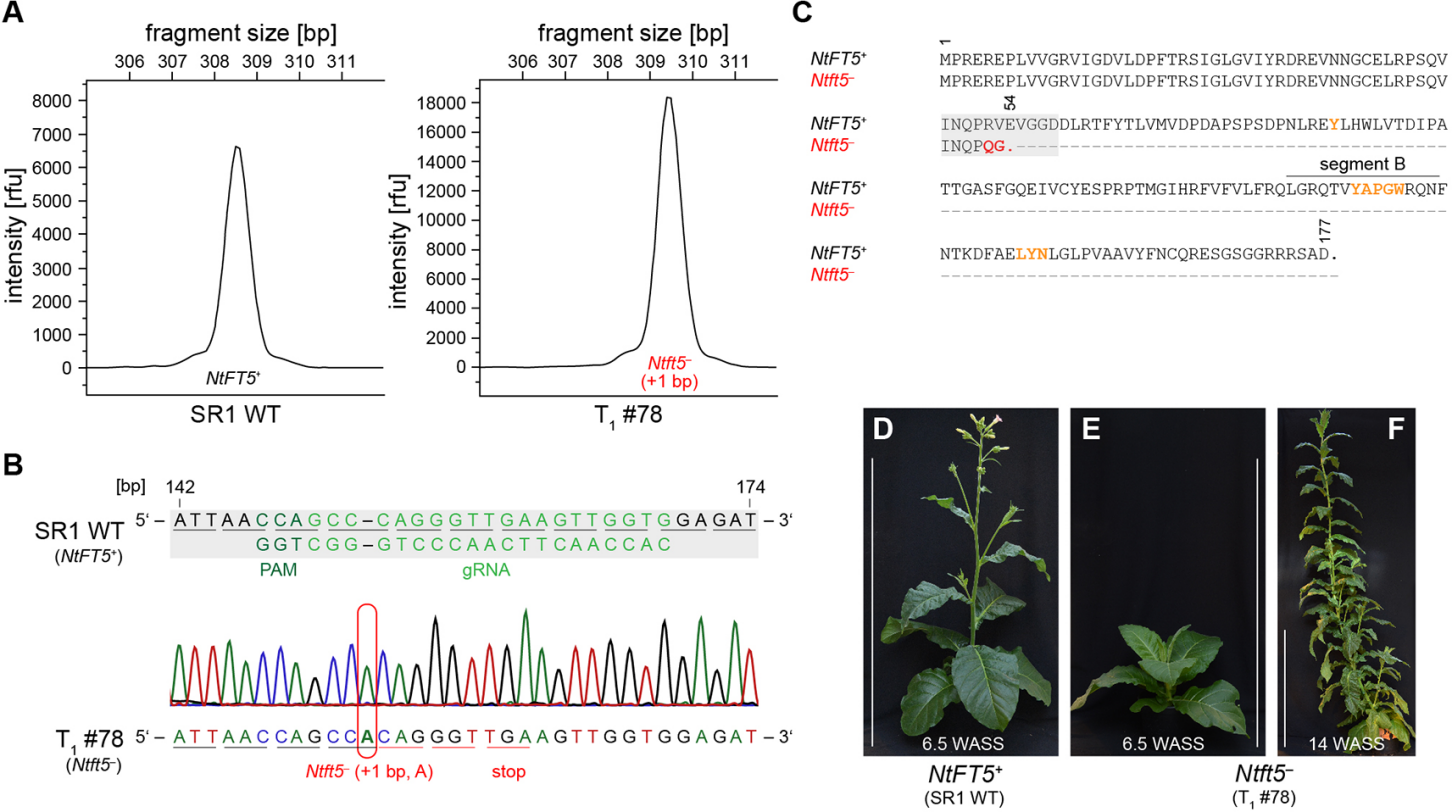
Characterization of *cas9*-free T_1_ plants under LD conditions. Analysis shown for the representative nullizygous knockout individual L2 #78 carrying two identical mutated *Ntft5^–^* alleles (+1 bp, A). **(A, B)** Genotyping of *NtFT5* was carried out by fragment length analysis **(A)** and direct PCR sequencing **(B)**. For both approaches, *NtFT5* was partly amplified by PCR using the primer combinations listed in **Supplemental Table S1**. **(A)** Fragment length analysis to investigate the *NtFT5* alleles. To identify allelic variants with indels, the electropherogram of the T_1_ plant was compared to the wild-type control. **(B)** Direct PCR sequencing of *NtFT5* in the T_1_ plant, showing partial alignment of the T_1_ plant chromatogram with the wild-type sequence (*NtFT5^+^*). The underlines indicate triplets corresponding to amino acids. **(C)**Alignment of native NtFT5 with its highly truncated protein version encoded by the *Ntft5^–^* allele. Amino acids required for the function of FT are highlighted in orange. **(D–F)** Phenotype of the T_1_ plant shown at the flowering stage of the wild-type control ~6.5 weeks after seed sowing (WASS) and ~14 WASS. Scale bars: 1 m.

The T_1_ generation of the self-pollinated L2 plant was cultivated under LD conditions and initially screened for the genomic integration of *cas9*. Beginning with seven *cas9*-free T_1_ plants (**Supplementary Figure S1B**), we determined the genotype in the mutated region of *NtFT5* by direct sequencing and amplicon fragment length analysis. All seven of the T_1_ plants only carried mutant *NtFT5* null alleles (+1 bp, A or C, hereafter referred to as *Ntft5^–^*), which we had already identified in the T_0_ generation (**Figure 2B**, **Supplementary Table S2**). The wild-type allele (hereafter, *NtFT5^+^*) was not present in any of these plants. As an example, T_1_ plant L2 #78 (**Figure 3**) was a homozygous knockout carrying two copies of the same mutant allele (+1 bp, A). The homozygous mutation in both alleles was confirmed by the uniform peak-shift of +1 bp in our fragment length analysis (**Figure 3A**) and this was resolved to the protospacer target site by direct PCR sequencing (**Figure 3B**). This insertion led to a highly-truncated protein of only 53 amino acids (**Figure 3C**), missing several key portions required for FT activity including segment B (Ahn et al., 2006; Ho and Weigel, 2014). In this individual, potential off-target mutations in selected *NtFT* genes were ruled out by amplifying and sequencing the corresponding part of exon I using the genomic PCR primers listed in **Supplementary Table S1**. The analysis was restricted to floral repressors *NtFT1*–*NtFT3*, floral activator *NtFT4*, as well as functionally-uncharacterized *NtFT8* and *NtFT12* because all other known *NtFT* genes in cv. SR1 are either non-functional or do not carry NGG-type or NRG-type PAMs at the potential off-target site (**Supplementary Figure S2**) (Harig et al., 2012; Beinecke et al., 2018). Most interestingly, phenotyping the T_1_ plants revealed that none of the nullizygous individuals could initiate flowering at the flowering stage of the wild-type cv. SR1 (**Figures 3D, E**). Along with the absence of flower induction at this time, the mutant plants also showed less elongation growth than the wild-type control. In the following weeks, however, the homozygous null *Ntft5^–^* plants grew rapidly during their vegetative phase, continuously generating biomass, and reaching ~3 m in height ~14 WASS (**Figure 3F**). Until this time, the plants showed no indication of floral bud formation. This ongoing vegetative growth resulted in the emergence of many additional leaves, comparable to plants overexpressing floral repressors *NtFT1–NtFT3* (Harig et al., 2012). Thus, the observed phenotype clearly showed that *NtFT5* is an essential floral activator under LD conditions.

### One Native *NtFT5* Allele is Sufficient to Restore Near-Normal Flowering

**Figure 4:**
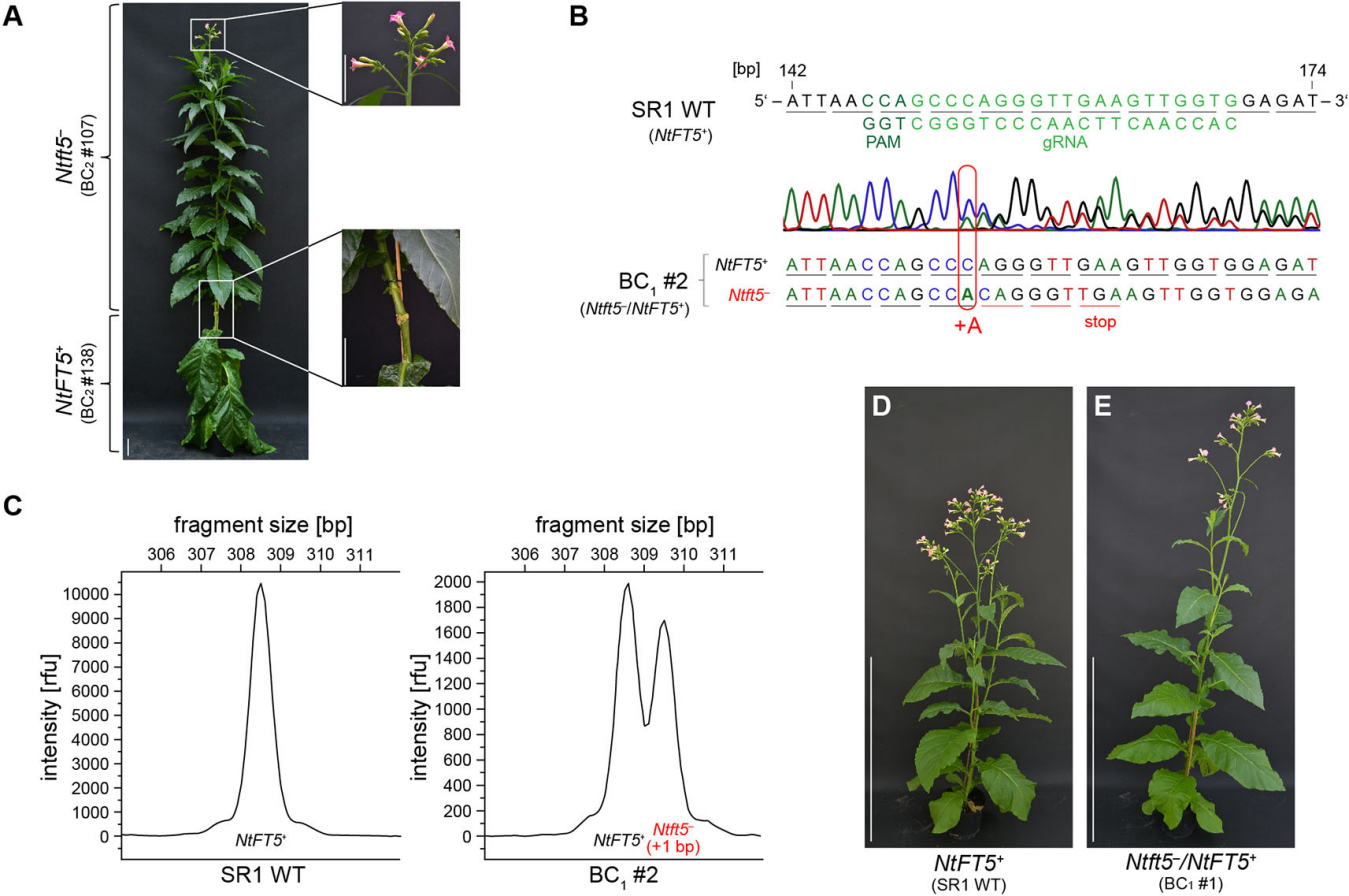
Flower induction of non-flowering nullizygous *Ntft5^–^* individuals by grafting and analysis of the first backcross generation (BC_1_) of nullizygous *Ntft5* ^–^ T_1_ plant #78 under LD conditions. **(A)** Grafting of a non-flowering nullizygous *Ntft5^–^* scion onto homozygous wild type *NtFT5^+^* rootstock and analysis under LD conditions. The representative grafting shown here was carried out using two randomly selected individuals of the *NtFT5* knockout BC_2_ generation ~8 weeks after seed sowing (WASS). Replacing the tip of the homozygous *NtFT5^+^* wild-type plant (#138), which had already developed floral buds, the vegetative main shoot of the nullizygous *Ntft5^–^* individual (#107) was grafted as a scion onto the wild-type rootstock. Documentation of the plants ~8 weeks after grafting, when the *Ntft5^–^* scion showed its first open flowers. Scale bars: 10 cm. (B–E) Analysis of heterozygous *Ntft5* ^–^/*NtFT5^+^* BC_1_ plants. **(B, C)** The *NtFT5* genotype of the BC_1_ plant was demonstrated by direct PCR sequencing **(B)** and fragment length analysis **(C)** using the primer combinations listed in **Supplementary Table S1** for PCR-based amplification. Genotyping of the representative heterozygous *Ntft5* ^–^/*NtFT5^+^* BC_1_ plant #2 is shown. **(B)** Partial alignment of *NtFT5* in the BC_1_ plant and the wild-type sequence (*NtFT5^+^*). The chromatogram of the BC_1_ plant was obtained by direct PCR sequencing of exon I. The underlines indicate triplets corresponding to amino acids. **(C)** Analysis of *NtFT5* allele fragment length. Mutated alleles carrying indels were identified by comparing the BC_1_ plant electropherogram with the wild-type control. **(D, E)** Phenotype of the representative heterozygous *Ntft5* ^–^/*NtFT5^+^* BC_1_ plant #1 shown in comparison to a wild-type control (*NtFT5^+^*) at the flowering stage ~8.5 weeks after seed sowing (WASS). Scale bar: 1 m.

To confirm that the observed non-flowering phenotype was caused by the knockout of *NtFT5*, we performed representative backcrossing experiments with T_1_ plant L2 #78 using wild-type cv. SR1 as a pollen donor. This strategy was only possible if the nullizygous *Ntft5* ^–^ T_1_ plants were able to flower, which we achieved by grafting the shoot as a scion onto wild-type rootstock (see **Figure 4A** for an example). Subsequent analysis of backcross generation 1 (BC_1_) was performed with a small number of plants under LD conditions (n = 4), shown in **Figure 4** for the representative BC_1_ plant. Genotyping revealed that the BC_1_ plants were heterozygous as expected (**Figures 4B, C**, and **Supplementary Table S3**). The coexistence of one mutant *Ntft5* ^–^ allele (+1 bp, A) and one wild-type *NtFT5^+^*allele was indicated by chaotic amplicon sequencing chromatograms downstream of the mutation site (**Figure 4B**) and was confirmed by the two major peaks in the fragment-length electropherograms (**Figure 4C**). Because all BC_1_ plants flowered (**Figures 4D, E**), it was apparent that the non-flowering phenotype of the parental homozygous null *Ntft5^–^* plant could be complemented by crossing, indicating that one native *NtFT5^+^* allele is sufficient for floral induction in cv. SR1. A more detailed phenotypic comparison of wild-type *NtFT5^+^*, heterozygous *Ntft5* ^–^/*NtFT5^+^* and nullizygous *Ntft5* ^–^ plants was conducted in BC_2_. For this purpose, one of the heterozygous BC_1_ plants (#2) was randomly chosen and self-pollinated for seed production.

### Heterozygous *NtFT5* Plants Carrying One Null Allele Show Beneficial Agronomic Traits

**Figure 5:**
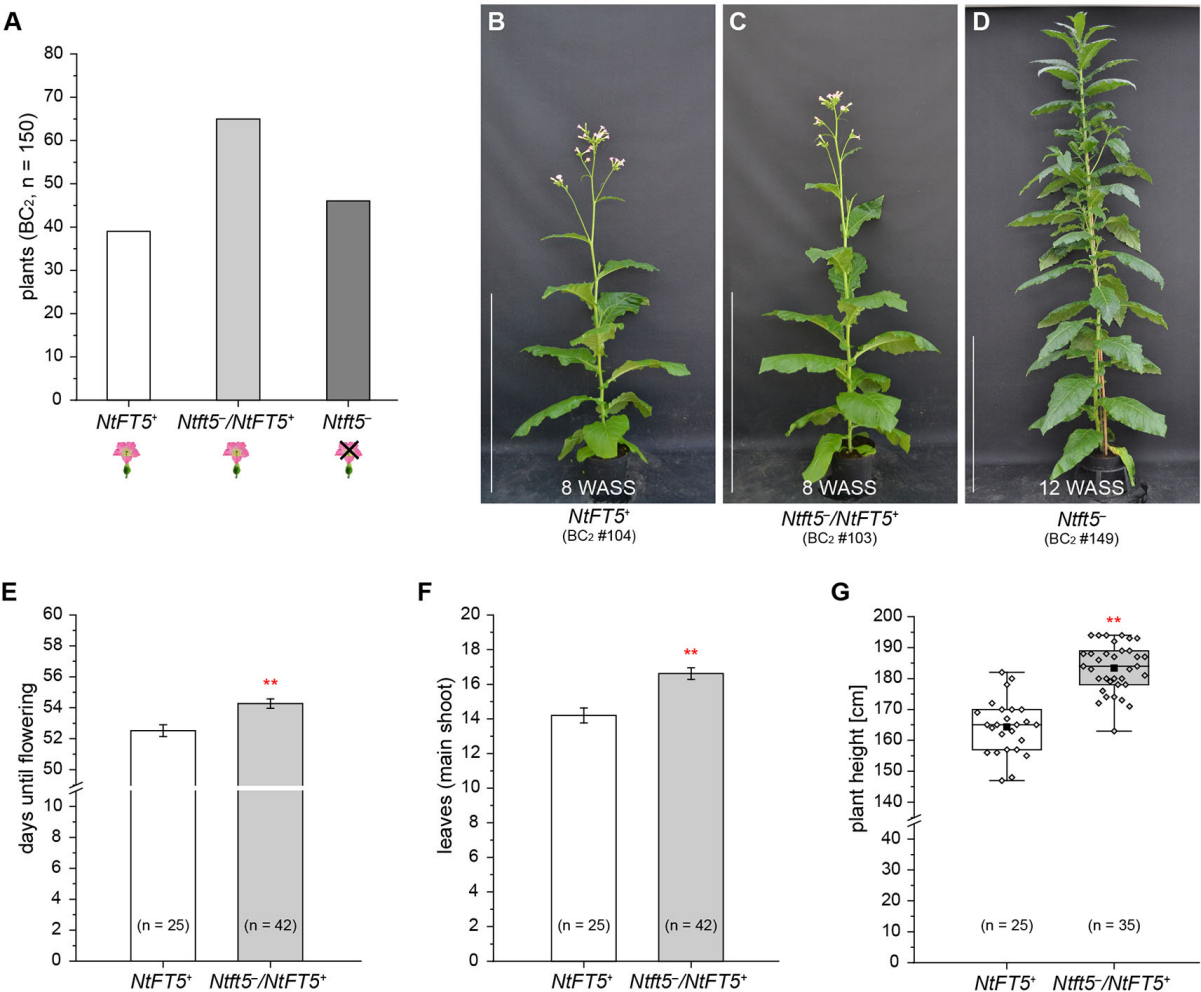
Detailed analysis of the second backcross generation (BC_2_, n = 150) of self-fertilized heterozygous *Ntft5* ^–^/*NtFT5^+^* BC_1_ plant #2 under LD conditions. For phenotyping, the BC_2_ plants (n = 150) were randomly split into two cultivation subgroups of 100 (group 1) and 50 (group 2) individuals, respectively. **(A)** Segregation of *NtFT5* genotypes in the original BC_2_ population. The plants were genotyped by fragment length analysis and direct PCR sequencing (**Supplemental Table S4**). **(B–D)** Phenotypes of wild-type *NtFT5^+^* and heterozygous *Ntft5^–^*/*NtFT5^+^* BC_2_ plants shown at an early flowering stage, when all of the individuals with these genotypes had already opened their first flowers ~8 weeks after seed sowing (WASS) in comparison to the vegetative phenotype of nullizygous *Ntft5^–^* plants ~12 WASS. Homozygous wild-type *NtFT5^+^* **(B)**, heterozygous *Ntft5^–^*/*NtFT5^+^* **(C)**, and nullizygous *Ntft5^–^* **(D)** plants are each represented by one individual from cultivation subgroup 2 as an example. Scale bars: 1 m. **(E–G)** Phenotypic comparison of homozygous *NtFT5^+^* and heterozygous *Ntft5* ^–^/*NtFT5^+^* BC_2_ individuals (subgroup 1) based on the days until flowering **(E)**, the number of leaves produced on the main shoot **(F)**, and the plant height **(G)**. The number of leaves and the plant height were determined simultaneously at an early flowering stage, ~8 weeks after seed sowing (WASS). **(E, F)** Mean values for the indicated sample sizes (n = 25–42) ± 95% confidence intervals shown as error bars. **(G)** The boxes delimit the 25^th^ to the 75^th^ percentiles of the datasets (n = 25–35). The median is illustrated as a horizontal line, the mean value as a filled square, and the individual measurements as diamonds. The lower and upper whiskers indicate values that differ least from the 25^th^ percentile – 1.5 ∙ IQR (interquartile range) or 75^th^ percentile + 1.5 ∙ IQR, respectively. **(E–G)** Normal distribution of the data was tested by applying the Kolmogorov-Smirnov test. Statistical significance was assessed by applying a pairwise Welch’s *t*-test (**P < 0.01).

Detailed phenotyping of the BC_2_ generation was performed with a large number of individuals (n = 150) under LD conditions. The genotypes of the BC_2_ plants were distributed as would be expected according to Mendelian segregation, with an approximate 1:2:1 ratio of homozygous *NtFT5* ^+^, heterozygous *Ntft5^–^*/*NtFT5^+^*, and nullizygous *Ntft5^–^* individuals (**Figure 5A** and **Supplementary Table S4**). In line with our earlier observations, all homozygous *NtFT5^+^* and heterozygous *Ntft5* ^–^/*NtFT5^+^*plants flowered (representative individuals are shown in **Figure 5B, C**). In contrast, all nullizygous *Ntft5^–^* plants (**Figure 5D**) were again unable to produce flowers at the wild-type flowering stage and until at least ~15 WASS. These segregation ratios suggest the absence of epistatic effects or other genetic interactions with the *NtFT5* genotype.

**Figure 6:**
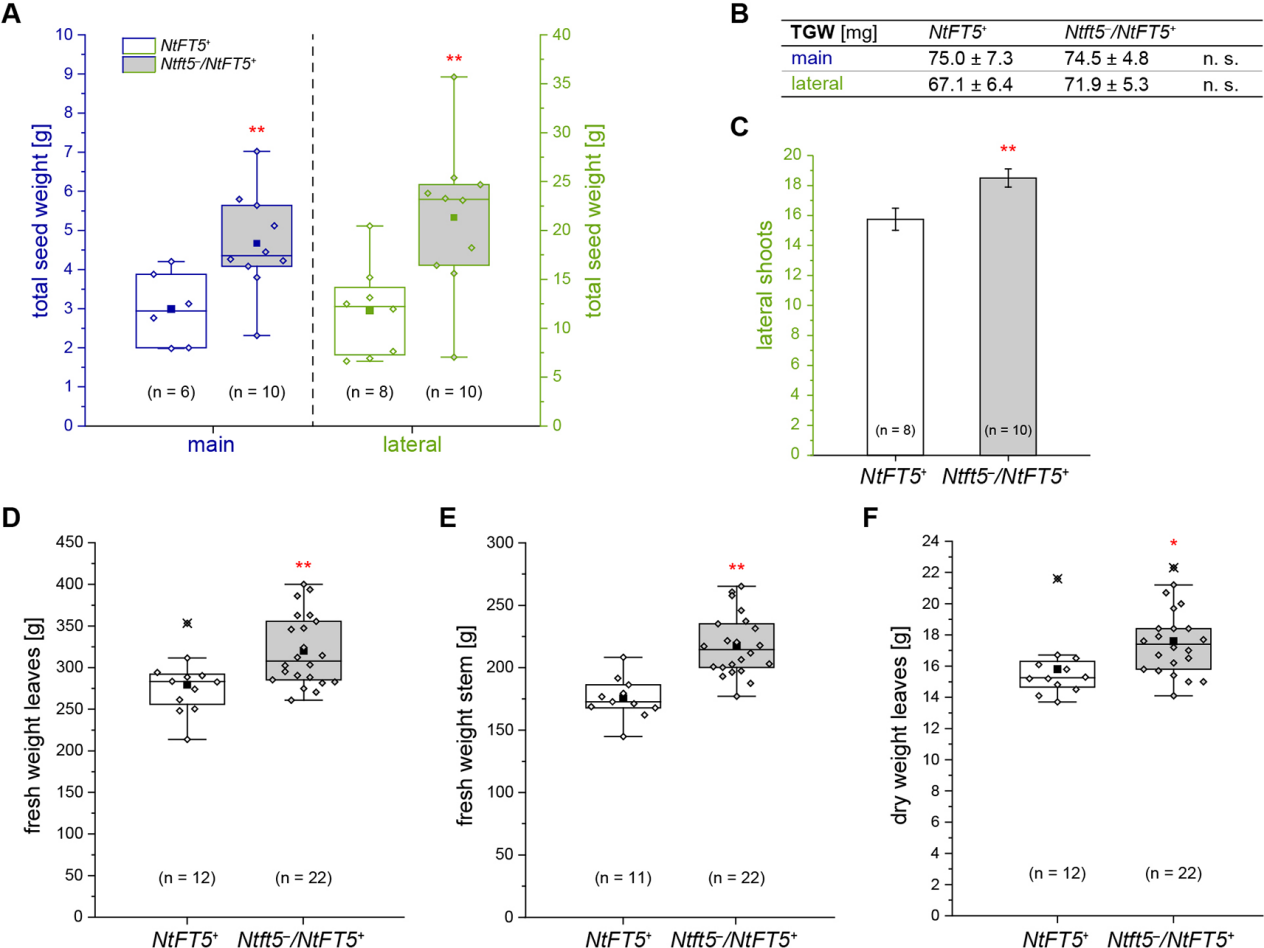
Detailed phenotyping of homozygous *NtFT5^+^* and heterozygous *Ntft5* ^–^/*NtFT5^+^* BC_2_ plants focusing on seed production and the generation of vegetative biomass. **(A–C)** Randomly selected BC_2_ individuals of both genotypes (cultivation subgroup 1) were analyzed to determine the total seed dry weight **(A)**, the thousand grain weight (TGW) of the seeds produced by main and lateral shoots **(B)**, and the number of lateral shoots **(C)**. **(A, B)** For quantification of the seed weight, the capsules produced by the main and lateral shoots were simultaneously harvested ~15 weeks after seed sowing (WASS). The seeds were dried, removed from the capsules, and the total weight determined. TGW measurement was carried out in technical duplicates and the replicates used to calculate the mean. **(C)** The number of lateral shoots was determined ~13.5 WASS. **(B, C)** Mean values of the indicated sample sizes (n = 8–10) ± 95% confidence intervals for means (shown in **(C)** as error bars). **(D–F)** Individuals in the second cultivation subgroup were characterized to determine the vegetative biomass produced on the main shoot. The total fresh weight of the leaves **(D)** and stem **(E)** was measured immediately after harvesting ~8 WASS, when all plants had already opened their first flowers. **(F)** The dry weight was determined after drying in a heating cabinet. **(A, D–F)** The boxes delimit values of the 25^th^ to the 75^th^ percentiles of the datasets (n = 6–22). The median is shown as a horizontal line, the mean as a filled square, and the individual measurements as diamonds. Outlier values are crossed out. The lower and upper whiskers indicate values that differ least from the 25^th^ percentile – 1.5 ∙ IQR (interquartile range) or 75^th^ percentile + 1.5 ∙ IQR, respectively. **(A–F)** Normal distribution of the data was tested by applying the Kolmogorov-Smirnov test. Statistical significance was assessed by applying a pairwise Welch’s *t*-test (**P < 0.01; *P < 0.05; n. s., not significant).

To assess the potential agronomic benefits of the heterozygous plants, we compared them with homozygous *NtFT5^+^* controls. The original population of 150 BC_2_ plants was randomly split into two cultivation subgroups for phenotyping. Homozygous *NtFT5^+^* and heterozygous individuals assigned to the first cultivation subgroup (n = 100, #1–100) were analyzed to determine the precise time point of flowering. We also counted the number of leaves and measured the plant height, both determined simultaneously at an early flowering stage when all plants had opened their first flowers (~8 WASS, **Figures 5E–G**). We observed a clear genotype-dependent effect on these three parameters. The heterozygous plants flowered with a slight mean delay of ~2 days (**Figure 5E**). This short extension of the vegetative growth phase resulted in the production of ~2 more leaves on the main shoot (**Figure 5F**), and the plants also grew higher (**Figure 5G**). To determine whether these observed differences affected important agronomic traits, we selected a smaller number of randomly chosen individuals from the first batch and cultivated them for a longer period of time until they generated ripened capsules ~15 WASS. At that time point, we simultaneously harvested all the capsules, separating those from the main and lateral shoots. After drying, the seeds were removed from the capsules to determine their total seed weight and thousand grain weight (TGW). We found that the *Ntft5* ^–^/*NtFT5^+^* plants produced a higher mean total seed weight from both the main and lateral shoots (**Figure 6A**). However, there was no significant difference in the mean TGW (**Figure 6B**), indicating that the higher total seed weight of the heterozygous plants reflected the greater number of seeds. For the seeds produced by lateral shoots, this phenomenon may reflect the greater abundance of those shoots (**Figure 6C**).

The second group of plants (n = 50, #101–150) was used to assess the impact of the extended vegetative growth phase on biomass generation. Therefore, homozygous *NtFT5^+^* and heterozygous plants were cultivated until all individuals generated their first flowers (~8 WASS), allowing us to quantify the amount of vegetative biomass on the main shoot, which is a particularly important agronomic trait. We separately harvested the leaves and the stem from each plant, leaving the main inflorescence, and measured the fresh weight of both tissues (**Figures 6D, E**). In the *Ntft5* ^–^/*NtFT5^+^*plants, we observed a significant increase in the mean fresh biomass weight of both the stem (~24%, **Figure 6D**) and the leaves (~14%, **Figure 6E**), which was mirrored by a similar trend in leaf dry weight (~11%, **Figure 6F**). These results are again likely to reflect the prolonged vegetative growth phase and greater abundance of leaves on the main shoot, as also observed in the first cultivation group (**Supplementary Figures S3A, B**). Our backcrossing experiments therefore clearly demonstrated that the heterozygous *Ntft5* ^–^/*NtFT5^+^* genotype provides agronomic benefits in terms of biomass and seed production compared to homozygous *NtFT5^+^*plants.

### Heterozygous *NtFT5* Plants Maintain Their Beneficial Agronomic Traits Under Various Forms of Abiotic Stress

The performance of crops is increasingly affected by climate change and it was therefore pertinent to determine whether the observed beneficial traits of heterozygous *Ntft5* ^–^/*NtFT5* ^+^ plants persist under stressful environmental conditions. Accordingly, heterozygous *Ntft5* ^–^/*NtFT5^+^* and homozygous *NtFT5^+^* BC_2_ plants were cultivated under LD conditions and were exposed to different forms of abiotic stress 30 days after seed sowing (DASS, **Table 1**). As above, we compared several key parameters (days until flowering, number of leaves, plant height, and the fresh and dry weight of leaves from the main shoot) of stressed heterozygous and homozygous BC_2_ plants when all plants under the same abiotic stress condition had opened their first flowers (**Table 1**).

For plants grown under salt stress, we observed similar beneficial traits to the plants grown under normal conditions. In the heterozygous plants, flowering was delayed by 4 days, ~3 more leaves were produced on the main shoot and the plants grew higher than homozygous plants (**Supplementary Figures 4A–C**). Additionally, the vegetative biomass in terms of fresh and dry weights of leaves on the main shoot were ~24% and ~30% higher, respectively, compared to homozygous plants (**Supplementary Figures 4D, E**). Our experiments thus demonstrated that salt stress does not diminish the enhanced biomass production of heterozygous *Ntft5* ^–^/*NtFT5^+^* plants compared to homozygous *NtFT5^+^* plants.

**Figure 7:**
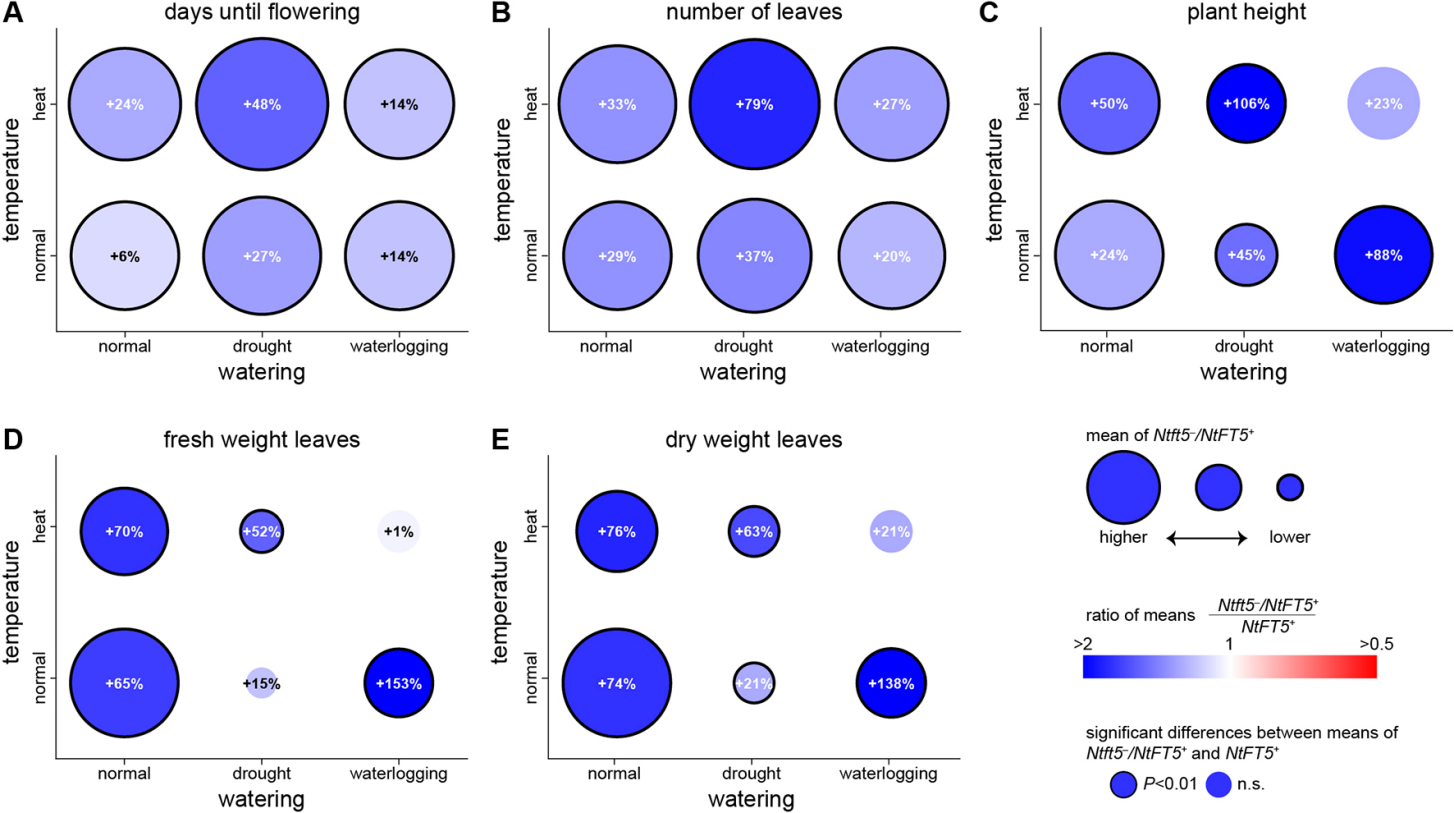
Phenotype of heterozygous *Ntft5* ^–^/*NtFT5^+^* plants compared to homozygous *NtFT5^+^* plants under various environmental conditions. **(A–E)** Randomly selected BC_2_ individuals of both genotypes were cultivated in the greenhouse under LD conditions at normal temperature in combination with normal watering, drought stress or waterlogging (bottom row circles), as well as heat stress in combination with normal watering, drought stress or waterlogging (top row circles). Samples sizes per treatment and genotype were n = 8–10 (see **Supplementary Figures S5–S9** for details). Phenotyping was carried out when all plants grown under the same conditions had opened their flowers, as described in **Table 1**. The days until opening of the first flower **(A)**, the number of leaves generated on the main shoot **(B)**, the height of the main shoot at first flowering **(C)**, as well as fresh weight **(D)** and dry weight of leaves **(E)** generated on the main shoot were determined. Circle colors and percentage values reflect the difference between heterozygous *Ntft5* ^–^/*NtFT5^+^* and homozygous *NtFT5^+^* plants grown under the same conditions. Blue colors indicate that the observed mean was higher for heterozygous than homozygous plants, and a higher saturation corresponds to a higher ratio in favor of heterozygous individuals. Equal means of both genotypes are colored white, and red colors would indicate a higher mean for homozygous plants. Percentages within circles show the relative increase for the heterozygous *Ntft5* ^–^/*NtFT5^+^* plants in comparison to homozygous *NtFT5^+^* plants cultivated under the same conditions. A black circle border indicates that the observed difference between heterozygous and homozygous plants within the same condition was statistically significant (*P* < 0.01) as determined using Welch’s *t*-tests with Holm-Bonferroni correction (no boarder = not significant, n.s.) The circle areas represent mean values of heterozygous plants, and for each measured variable the scale of the circles is normalized to normal temperature/normal watering conditions.

To mimic the uncertain weather conditions associated with climate change, we also cultivated the plants under normal and hot temperature conditions each in combination with different irrigation regimes (**Table 1**). The heterozygous *Ntft5* ^–^/*NtFT5^+^* plants maintained their superior performance under most of these conditions (**Figure 7**, for detailed comparisons see **Supplementary Figures 5–9**). We were able to reproduce the near-normal flowering of heterozygous plants under normal LD conditions, but the difference in flowering time points between *Ntft5* ^–^/*NtFT5^+^* and *NtFT5^+^* individuals was exaggerated when the plants were placed under stress (**Figure 7A**, **Supplementary Figure S5**). The delayed flowering of heterozygous plants compared to the *NtFT5^+^* individuals ranged from a minimum of ~7 days under normal temperature or heat stress conditions when each was combined with waterlogging, to a maximum of ~27 days for the combination of heat and drought stress. Moreover, the heterozygous plants consistently produced more leaves across all tested conditions, especially when exposed to the combination of heat and drought stress (**Figure 7B**, **Supplementary Figure S6**). Under all the test conditions, and most notably under drought stress, the homozygous plants showed a stunted phenotype due to the shortening of the shoot to varying degrees (**Supplementary Figure S7**). This phenomenon coincided with a clear reduction in leaf biomass on the main shoot compared to control plants of the same genotype grown under normal LD conditions (**Supplementary Figures S8** and **S9**). The heterozygous plants were consistently taller than the homozygous plants with the exception of those grown under combined heat stress and waterlogging conditions (**Figure 7C**, **Supplementary Figure S7**). Most strikingly, the shoots of the *Ntft5* ^–^/*NtFT5^+^* plants were approximately twice as long as the *NtFT5^+^* plants grown under normal temperature conditions with waterlogging, or under a combination of heat and drought stress.

Even under normal LD conditions, the heterozygous *Ntft5* ^–^/*NtFT5^+^* plants produced 65% more fresh leaf biomass and 74% more dry leaf biomass on the main shoot than homozygous *NtFT5^+^* plants (**Figures 7D, E** and **Supplementary Figures S8** and **S9**). In contrast to growth under normal LD conditions, the heterozygous plants generated only 21% more dry leaf biomass compared to the homozygous plants under normal temperature conditions with drought stress, and heat stress combined with waterlogging (statistically nonsignificant; **Figure 7E**). Nevertheless, under heat stress with normal watering, and under combined heat and drought stress, the heterozygous plants were still able to produce 76% and 63% more dry leaf biomass, respectively. The most striking differences between heterozygous *Ntft5* ^–^/*NtFT5^+^* and *NtFT5^+^* plants were observed under waterlogging conditions, where the heterozygous plants produced 138% more dry leaf biomass. The stress experiments therefore clearly showed that the heterozygous plants not only produce more biomass than the homozygous plants under normal conditions, but also under most forms of abiotic stress.

## Discussion

The agronomic properties of today’s crops must be improved to optimize the use of available agricultural land in order to provide for the growing world population and address the challenges of climate change. Research has therefore focused on regulators of fundamental developmental processes in plants, such as flowering, allowing the establishment of new breeding programs (e.g., Jung and Müller, 2009). Members of the *FT*-like gene family are key regulators of flowering in diverse plant species, and are therefore common targets for crop improvement (e.g., de Bossoreille de Ribou et al., 2013; Wickland and Hanzawa, 2015).

In the model crop *N. tabacum*, five *FT*-like genes (*NtFT1–NtFT5*) have been characterized thus far. *NtFT1*–*NtFT3* encode floral repressors, whereas *NtFT4* and *NtFT5* encode floral inducers (Harig et al., 2012; Beinecke et al., 2018; Wang et al., 2018). Furthering our earlier studies, here we assessed the potential of *NtFT5* as target for breeding programs by investigating the expression and function of the gene in detail (Harig et al., 2012; Beinecke et al., 2018). We analyzed the expression of *NtFT5* in the leaves and stem of SR1 plants by qPCR and the detection of reporter gene products driven by the P*_NtFT5_* promoter. Like the other tobacco *FT* genes, we found that *NtFT5* was expressed in the phloem of leaves, but we also observed strong expression in the phloem of the stem, which is not the case for any other *NtFT* gene characterized thus far (Harig et al., 2012; Beinecke et al., 2018). The abundance of *NtFT5* mRNA in the stem clearly indicated this tissue as a major site of expression, and the specific restriction of *NtFT5* expression to the phloem companion cells confirms the predicted role of the corresponding protein as a mobile stimulus.

Despite slight differences in transcript levels under SD and LD conditions, *NtFT5* expression is largely photoperiod-independent not only in the leaves but also in the stem. In at least the apical leaves and stem, we also found an increase in *NtFT5* expression during the transition from vegetative growth to reproduction. In SR1 plants, this tendency was also observed for the other *NtFT* genes under SD conditions in the leaves (Harig et al., 2012). This suggests that during short days all of these genes are probably involved in the regulation of flowering in concert. However, given our previous findings that *NtFT5* silencing delays flowering under both LD and SD conditions, our results indicate that *NtFT5* is the dominant floral activator in *N. tabacum*, and its day-neutral abundance in the stem suggests that it has diverged from the photoperiod-dependent and leaf-specific expression of *NtFT1–NtFT4* (Harig et al., 2012; Beinecke et al., 2018).

To assess the role of NtFT5 in floral induction in more detail, we used the CRISPR/Cas9 system to knock out the *NtFT5* gene in *N. tabacum* SR1 plants. Knockout mutants lacking the *cas9* transgene and carrying two null *Ntft5^–^* alleles were unable to flower under LD conditions, highlighting the importance of *NtFT5* as a floral activator in this agriculturally-relevant scenario. Due to the persistent vegetative phenotype of the *NtFT5* knockout plants, it is clear that other floral activators cannot compensate for the loss of NtFT5 under LD conditions. In contrast, such plants may still be able to flower under SD conditions due to the presence of NtFT4 (Harig et al., 2012).

We were able to induce flowering in the knockout plants even under LD conditions by grafting homozygous null *Ntft5* ^–^ scions onto wild-type rootstock followed by the application of pollen from a wild-type donor. Given that NtFT5 is expressed specifically in phloem companion cells, this provides further evidence that NtFT5 functions as a mobile signal that can cross graft junctions to induce flowering at the shoot tip of the grafted plants, as previously described for the mobility of FT in *A. thaliana* (Corbesier et al., 2007; Mathieu et al., 2007). The companion cell-specific expression allows the long-distance transport of FT proteins through the phloem, fulfilling the function of a systemic florigen (Corbesier et al., 2007; Mathieu et al., 2007; Tamaki et al., 2007; Komiya et al., 2009).

In contrast to the nullizygous *Ntft5* ^–^ plants, heterozygous *Ntft5* ^–^/*NtFT5^+^* backcross generation plants demonstrated a near-normal flowering phenotype, clearly showing that one wild-type *NtFT5* allele is sufficient for floral induction. However, the heterozygous plants flowered on average ~2 days later than their wild-type counterparts, producing more leaves on the main shoot. About a century ago, a similar phenomenon was observed when giant Mammoth tobacco mutants were crossed with wild-type plants, yielding F_1_ hybrids that flowered in a manner similar to the wild-type control but grew taller and produced more leaves (Allard, 1919).

Detailed phenotypic comparison of homozygous *NtFT5* ^+^ and heterozygous *Ntft5* ^–^/*NtFT5^+^* plants under LD conditions demonstrated clearly that the short extension of the vegetative growth phase conferred beneficial agronomic traits. In addition to a higher total quantity of seeds, *Ntft5* ^–^/*NtFT5^+^* plants generated significantly more vegetative biomass on the main shoot during the same cultivation period. Agronomic benefits have already been reported for *FT*-heterozygous plants in the case of the tomato *SINGLE FLOWER TRUSS* (*SFT*) gene, where *sft^–^/SFT^+^* heterozygotes in a certain genetic background produced higher fruit yields than homozygous *SFT^+^* individuals. Similar to our case, the lower dosage of *FT* briefly prolonged the vegetative growth phase in sympodial shoots, resulting in the development of more leaf nodes and inflorescences (Krieger et al., 2010).

We also demonstrated that heterozygous *Ntft5* ^–^/*NtFT5^+^* plants retained their beneficial agronomic traits under various abiotic stress conditions (i.e., they grew taller and produced more vegetative biomass on the main shoot) compared to homozygous *NtFT5^+^* plants. Plants that show greater resistance or tolerance towards changing environmental conditions will be indispensable to maintain crop productivity in the face of global climate change. Tobacco plants exposed to temperatures only 5°C higher than normal cultivation conditions are stunted and leaf expansion is inhibited (Yang et al., 2018). In our experiments, heterozygous *Ntft5* ^–^/*NtFT5^+^* plants grown at temperatures up to 15°C above normal cultivation conditions (**Table 1**) generated similar amounts of biomass as homozygous *NtFT5^+^* plants cultivated under normal conditions (**Figures 7D, E** and **Supplementary Figures S8** and **S9**). Given that the heterozygous *Ntft5* ^–^/*NtFT5^+^* plants perform well under various abiotic stress conditions, FT homologs that act as floral inducers are likely to be promising candidates for breeding programs aiming to adapt crops to the effects of climate change. It would be intriguing to attempt this strategy in vegetative crops such as potato, sugar beet or lettuce, where the allocation of energy to vegetative organs is desirable and a prolonged vegetative growth phase achieved by modulating the activity of FT homologs could therefore be advantageous. The potato StSP3D protein is an FT-like floral activator, and silencing the corresponding gene in *S. tuberosum* ssp. *andigenum* genotypes can delay or completely inhibit flowering, but still permits tuberization (Navarro et al., 2011). However, tuberization is induced by another FT homolog (StSP6A) and it is postulated that allelic variation in the *StSP6A* pathway is closely associated with potato domestication in different latitudes with corresponding differences in day length (Kloosterman et al., 2013; Morris et al., 2014). Therefore, the modulation of StSP3D levels to prolong vegetative growth in combination with these beneficial tuberization alleles could increase the yield of potato crops even further. For sugar beet and lettuce, the expression of FT-like floral activators is closely linked with the undesirable phenomenon of bolting (Pin et al., 2010; Chen et al., 2018). The depletion of *BvFT2* or *LsFT* mRNA by RNA interference causes delayed bolting, suggesting that both genes could be ideal candidates for crop improvement *via* conventional breeding to reduce *FT* dosage.

In agreement with our previous report that *NtFT5* overexpression leads to early flowering and silencing delays it (Beinecke et al., 2018), we found that the abundance of *NtFT5* mRNA has a decisive impact on flowering time, allowing the modulation of vegetative versus reproductive growth by targeting *NtFT5* expression levels. *NtFT5* is therefore a promising target for future breeding programs. Early-flowering tobacco plants overexpressing *NtFT5* could be useful laboratory models because the generation time is shorter than normal (Beinecke et al., 2018; Wang et al., 2018). In contrast, heterozygous *Ntft5* ^–^/*NtFT5^+^* plants would be useful in an agronomic context because they produce more biomass than wild-type plants during the same cultivation period. Finally, the nullizygous *Ntft5* ^–^ plants may be induced to flower by grafting or cultivation under SD conditions to produce seeds. Their offspring could then be trapped in the vegetative phase by cultivation under LD conditions, allowing the massive production of biomass without any risk of outcrossing. Taken together, our results provide a more detailed understanding of floral induction in the day-neutral plant *N. tabacum*, revealing that NtFT5 is an essential regulator of flowering under agriculturally-relevant LD conditions and showing conclusively that FT homologs are promising targets for breeding programs in other plant species, especially in biomass crops.

## Data Availability Statement

The raw data supporting the conclusions of this article will be made available by the authors, without undue reservation, to any qualified researcher.

## Author Contributions

FS, MZ, LG, JM, DP, and GN conceived and designed the experiments. FS, MZ, and SL conducted the experiments. FS, MZ, DW, and RT analyzed the data. DP and GN contributed the reagents, materials, and analytical tools. FS and MZ wrote the manuscript. All authors helped to revise the manuscript and approved the submitted version.

## Funding

This work was partially supported by grants from the Fraunhofer Gesellschaft, Munich, Germany.

## Conflict of Interest

The authors declare that the research was conducted in the absence of any commercial or financial relationships that could be construed as a potential conflict of interest.
