# Supplementary material for: The Major Floral Promoter NtFT5 in Tobacco (Nicotiana tabacum) Is a Promising Target for Crop Improvement
Source: Front Plant Sci. 2020 Jan 10;10:1666. doi: 10.3389/fpls.2019.01666 (PMC6966700; doi:10.3389/fpls.2019.01666)
Supplement: Supplementary file 1 [file DataSheet_1.pdf]

Supplementary Material

The Major Floral Promoter NtFT5 in Tobacco (*Nicotiana tabacum*) is a Promising Target for Crop Improvement

Florentin J. Schmidt[1♦], Marius M. Zimmermann[1♦], David R. Wiedmann[2], Sophie Lichtenauer[1], Lena Grundmann[2], Jost Muth[3], Richard M. Twyman[4], Dirk Prüfer[1,2] and Gundula A. Noll[1*]

[1]Institute of Plant Biology and Biotechnology, University of Münster, Münster, Germany
[2]Fraunhofer Institute for Molecular Biology and Applied Ecology IME, Münster, Germany
[3]Fraunhofer Institute for Molecular Biology and Applied Ecology IME, Aachen, Germany, and
[4]TRM Ltd, PO Box 493, Scarborough YO11 9FJ, UK

♦ These authors contributed equally to this work.

*** Correspondence:**
Gundula A. Noll
gnoll@uni-muenster.de

Keywords: **FLOWERING LOCUS T (FT), SR1, floral activator, long-day flowering, PEBP family, CRISPR/Cas9, loss-of-function-mutation, phloem companion cell-specific expression**

Supplementary Data

Figure S1 | PCR-based screening of genomic *cas9* integration in T_0 and T_1 plants of the nullizygous *NtFT5*$^{-}$ line.

Figure S2 | Partial sequence alignment of the *NtFT5* coding sequence with the corresponding regions in *NtFT1*–*NtFT13*.

Figure S3 | Flowering phenotypes of homozygous *NtFT5*$^{+}$ and heterozygous *Ntft5*$^{-}$/*NtFT5*$^{+}$ second backcross generation (BC_2) plants of self-fertilized BC_1 plant #2 cultivated in the second subgroup (n = 50).

Figure S4 | Detailed phenotypic analysis of salt-stressed homozygous *NtFT5*$^{+}$ and heterozygous *Ntft5*$^{-}$/*NtFT5*$^{+}$ second backcross generation (BC_2) plants grown under LD conditions.

Figure S5 | Days until flowering of homozygous *NtFT5*$^{+}$ and heterozygous *Ntft5*$^{-}$/*NtFT5*$^{+}$ second backcross generation (BC_2) plants cultivated under various environmental LD conditions.

Figure S6 | Number of leaves of homozygous *NtFT5*$^{+}$ and heterozygous *Ntft5*$^{-}$/*NtFT5*$^{+}$ second backcross generation (BC_2) plants cultivated under various environmental LD conditions.

Figure S7 | Height of homozygous *NtFT5*$^{+}$ and heterozygous *Ntft5*$^{-}$/*NtFT5*$^{+}$ second backcross generation (BC_2) plants cultivated under various environmental LD conditions.

Figure S8 | Leaf fresh weight of homozygous *NtFT5*$^{+}$ and heterozygous *Ntft5*$^{-}$/*NtFT5*$^{+}$ second backcross generation (BC_2) plants cultivated under various environmental LD conditions.

Figure S9 | Leaf dry weight of homozygous *NtFT5*$^{+}$ and heterozygous *Ntft5*$^{-}$/*NtFT5*$^{+}$ second backcross generation (BC_2) plants cultivated under various environmental LD conditions.

Table S1 | List of primers used in this study.

Table S2 | Genotypes of the seven identified *cas9*-free *NtFT5*-knockout plants (T_1 generation of L2).

Table S3 | Plant genotypes of the first backcross generation (BC_1, n = 4) of backcrossed nullizygous *Ntft5*$^{-}$ T_1 plant L2 #78.

Table S4 | Plant genotypes of the second backcross generation (BC_2, n = 150) of self-fertilized heterozygous *Ntft5*$^{-}$/*NtFT5*$^{+}$ BC_1 plant #2.

Table S5 | GenBank accession numbers of *NtFT1*–*NtFT13* coding sequences.

1 Supplementary Figures

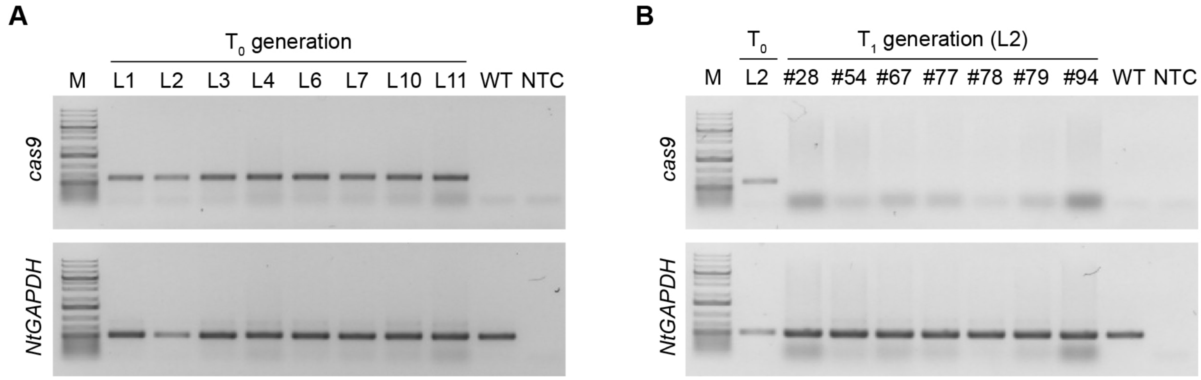

Figure S1 | PCR-based screening of genomic *cas9* integration in T_0 and T_1 plants of the nullizygous *NtFT5*$^{-}$ line L2. Analysis of independently-regenerated transgenic T_0 lines **(A)** and *cas9*-free T_1 plants of L2 **(B)**. PCR amplification of *cas9* and *N. tabacum glyceraldehyde-3-phosphate dehydrogenase* (*NtGAPDH*, template control) was carried out using genomic DNA and the gene specific primer combinations listed in Supplementary Table S1. The genomic DNA of *N. tabacum* wild-type cv. SR1 plants (WT) was used as negative control and a no template control (NTC) was also tested. The PCR products were separated by 1% (w/v) agarose gel electrophoresis, with the Gene Ruler 1 kb DNA Ladder (Thermo Fisher Scientific) used as size markers (M).

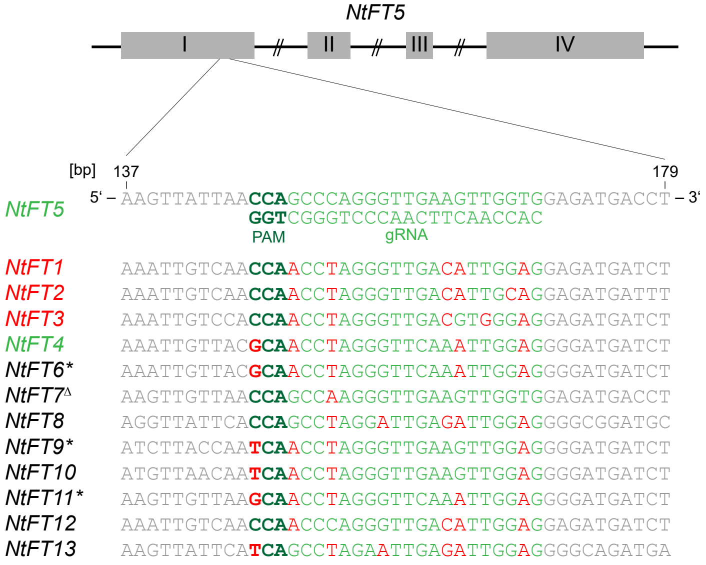

Figure S2 | Partial sequence alignment of the *NtFT5* coding sequence with the corresponding regions in *NtFT1–NtFT13*. The alignment shows the location of the *NtFT5*-specific protospacer and the protospacer adjacent motif (PAM) on the antisense DNA strand in the 147–169 bp region of exon I (for details, see Figure 2A). Gene names in green = known floral inducers; red = known floral repressors; black = non-functional genes (in *N. tabacum* cv. SR1) and genes with unknown function. Asterisks indicate premature stop codons. Triangles indicate genes not present in *N. tabacum* cv. SR1 (Beinecke et al., 2018). Mismatches compared to the *NtFT5* sequence are highlighted in red. The GenBank accession numbers corresponding to the *NtFT1–NtFT13* coding sequences (Harig et al., 2012; Beinecke et al., 2018) are listed in Supplementary Table S5.

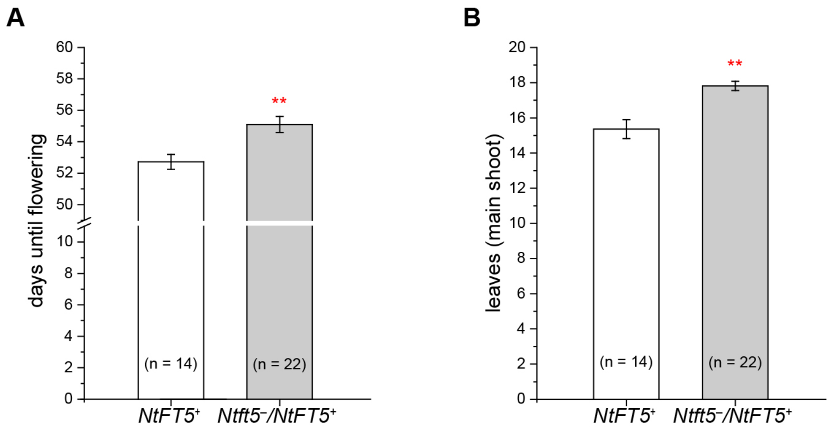

Figure S3 | Flowering phenotypes of homozygous *NtFT5*$^{+}$ and heterozygous *Ntft5*$^{-}$/*NtFT5*$^{+}$ second backcross generation (BC_2) plants of self-fertilized BC_1 plant #2 cultivated in the second subgroup (n = 50). **(A)** Days until flowering. **(B)** Number of leaves produced on the main shoot. **(A, B)** The number of leaves was determined simultaneously at an early flowering stage ~8 weeks after seed sowing (WASS). Mean values represent the indicated sample sizes (n = 14–22) ± 95% confidence intervals for means shown as error bars. Normal distribution of the data was tested by applying the Kolmogorov-Smirnov test. Statistical significance was assessed by applying a pairwise Welch's *t*-test (**P<0.01).

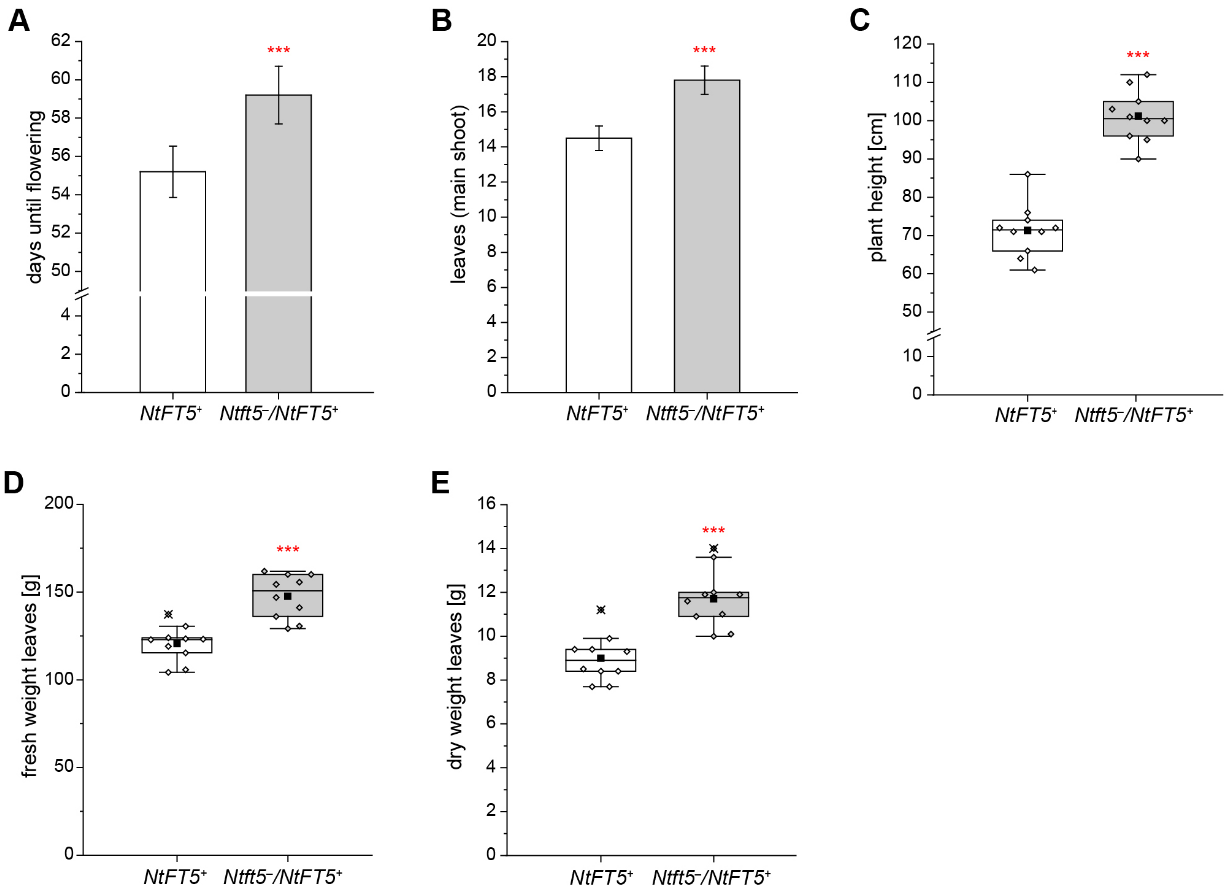

Figure S4 | Detailed phenotypic analysis of salt-stressed homozygous *NtFT5*$^+$ and heterozygous *Ntft5*$^-$/*NtFT5*$^+$ second backcross generation (BC_2) plants grown under LD conditions. Phenotypic comparison was based on the days until flowering **(A)**, number of leaves produced on the main shoot **(B)**, the plant height **(C)**, and the total fresh **(D)** and dry **(E)** weight of the leaves produced on the main shoot. The number of leaves, the plant height, and the leaf fresh weight were determined simultaneously when all plants had opened their first flowers 63 days after seed sowing. The leaf dry weight was determined after drying in a heating cabinet. **(A, B)** Bars indicate mean values (n = 10) and error bars ± 95% confidence intervals for means. **(C–E)** The boxes delimit the 25th to the 75th percentiles of the datasets (n = 10). The median is illustrated as a horizontal line, the mean value as a filled square, and the individual measurements as diamonds. The lower and upper whiskers indicate values that differ least from the 25th percentile – 1.5 · IQR (interquartile range) or 75th percentile + 1.5 · IQR, respectively. **(A–E)** Normal distribution of the data was tested by applying the Kolmogorov-Smirnov test. Statistical significance was assessed by applying a pairwise Welch's *t*-test (****P*<0.001).

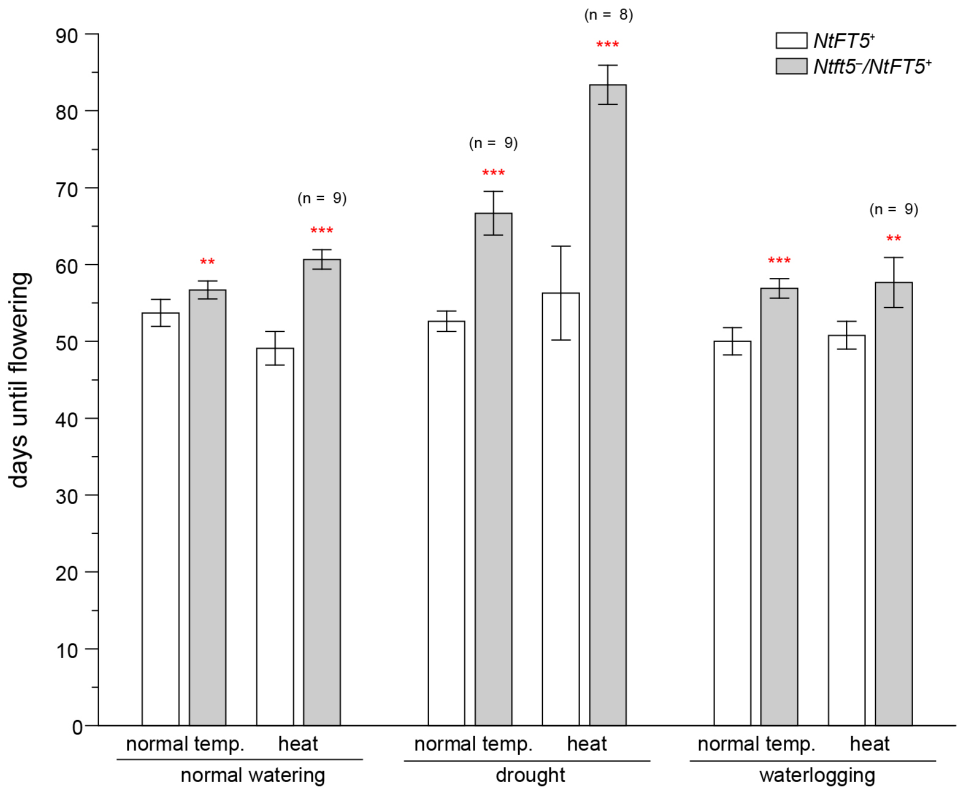

Figure S5 | Days until flowering of homozygous *NtFT5*$^+$ and heterozygous *Ntft5*$^–$/*NtFT5*$^+$ second backcross generation (BC_2) plants cultivated under various environmental LD conditions. Individuals were grown under standard LD conditions and under the influence of different abiotic stress treatments (for details, see Table 1). Bars indicate mean values (n = 10 unless stated otherwise) and error bars ± 95% confidence intervals for means. Normal distribution of the data was tested by applying the Kolmogorov-Smirnov test. For each treatment, the statistical significance of the difference between homozygous *NtFT5*$^+$ and heterozygous *Ntft5*$^–$/*NtFT5*$^+$ plants was assessed by applying Welch's *t*-test and *P*-values were adjusted with Holm-Bonferroni correction (****P*<0.001, ***P*<0.01).

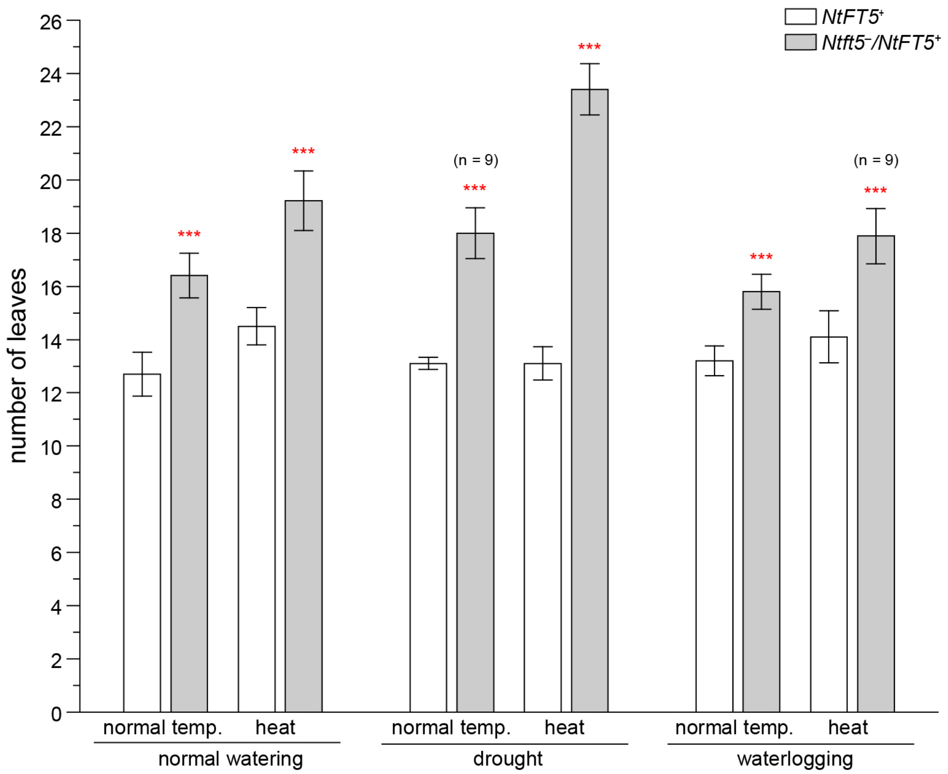

Figure S6 | Number of leaves of homozygous *NtFT5*$^{+}$ and heterozygous *Ntft5*$^{–}$/*NtFT5*$^{+}$ second backcross generation (BC_2) plants cultivated under various environmental LD conditions. Individuals were grown under standard LD conditions and under the influence of different abiotic stress treatments (for details, see Table 1). The number of leaves was determined simultaneously when all plants cultivated under the same conditions had opened their first flowers (Table 1). Bars indicate mean values (n = 10 unless stated otherwise) and error bars ± 95% confidence intervals for means. Normal distribution of the data was tested by applying the Kolmogorov-Smirnov test. For each treatment statistical significance of the difference between homozygous *NtFT5*$^{+}$ and heterozygous *Ntft5*$^{–}$/*NtFT5*$^{+}$ plants was assessed by applying Welch's *t*-test and *P*-values were adjusted with Holm-Bonferroni correction (****P*<0.001).

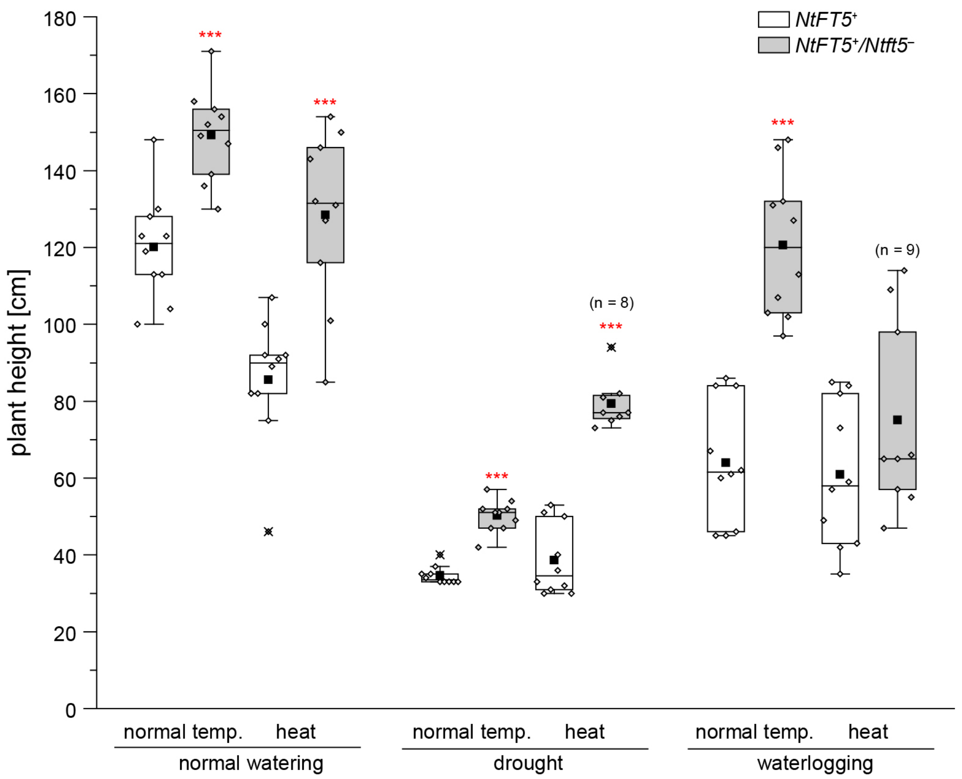

Figure S7 | Height of homozygous *NtFT5*$^{+}$ and heterozygous *Ntft5*$^{-}$/*NtFT5*$^{+}$ second backcross generation (BC_2) plants cultivated under various environmental LD conditions. Individuals were grown under standard LD conditions and under the influence of different abiotic stress treatments (for details, see Table 1). The plant height was determined simultaneously when all plants cultivated under the same conditions had opened their first flowers (Table 1). The boxes delimit the 25th to the 75th percentiles of the datasets (n = 10, unless stated otherwise). The median is illustrated as a horizontal line, the mean value as a filled square, and the individual measurements as diamonds. Outlier values are crossed out. The lower and upper whiskers indicate values that differ least from the 25th percentile – 1.5 · IQR (interquartile range) or 75th percentile + 1.5 · IQR, respectively. Normal distribution of the data was tested by applying the Kolmogorov-Smirnov test. For each treatment statistical significance of the difference between homozygous *NtFT5*$^{+}$ and heterozygous *Ntft5*$^{-}$/*NtFT5*$^{+}$ plants was assessed by applying Welch's *t*-test and *P*-values were adjusted with Holm-Bonferroni correction (****P*<0.001).

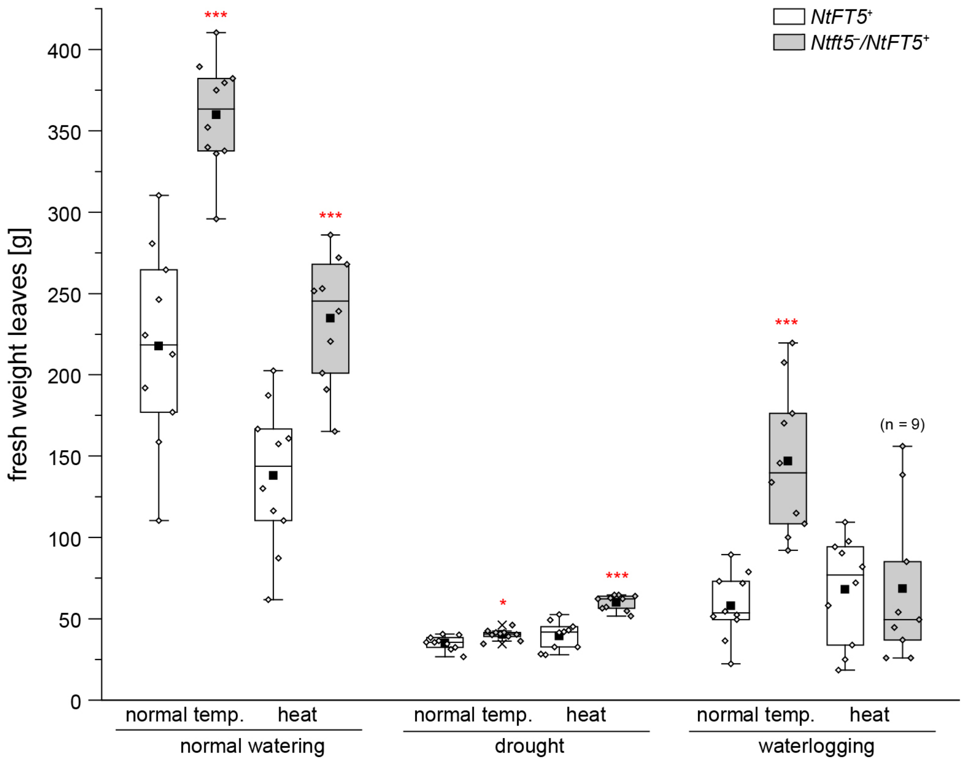

Figure S8 | Leaf fresh weight of homozygous *NtFT5*$^{+}$ and heterozygous *Ntft5*$^{-}$/*NtFT5*$^{+}$ second backcross generation (BC_2) plants cultivated under various environmental LD conditions. Individuals were grown under standard LD conditions and under the influence of different abiotic stress treatments (for details, see Table 1). The fresh weight of leaves on the main shoot was simultaneously determined when all plants grown under the same abiotic stress condition had opened their first flowers (Table 1). The boxes delimit the 25th to the 75th percentiles of the datasets (n = 10, unless stated otherwise). The median is illustrated as a horizontal line, the mean value as a filled square, and the individual measurements as diamonds. Outlier values are crossed out. The lower and upper whiskers indicate values that differ least from the 25th percentile – 1.5 · IQR (interquartile range) or 75th percentile + 1.5 · IQR, respectively. Normal distribution of the data was tested by applying the Kolmogorov-Smirnov test. For each treatment statistical significance of the difference between homozygous *NtFT5*$^{+}$ and heterozygous *Ntft5*$^{-}$/*NtFT5*$^{+}$ plants was assessed by applying Welch's *t*-test and *P*-values were adjusted with Holm-Bonferroni correction (***P<0.001; *P<0.05).

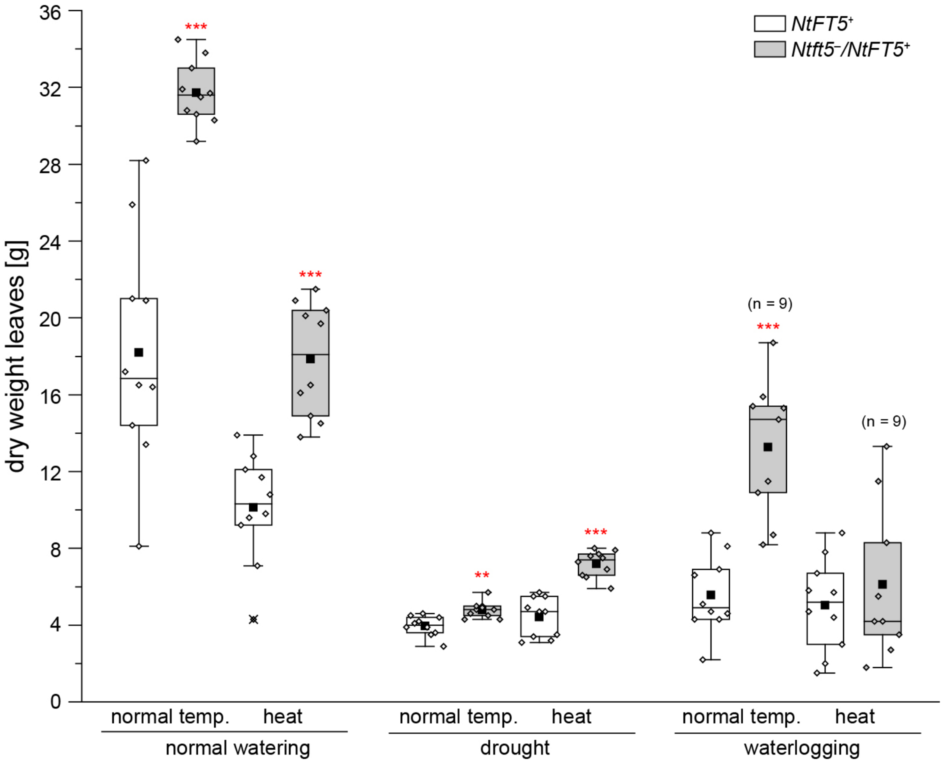

Figure S9 | Leaf dry weight of homozygous *NtFT5*$^{+}$ and heterozygous *Ntft5*$^{-}$/*NtFT5*$^{+}$ second backcross generation (BC_2) plants cultivated under various environmental LD conditions. Individuals were grown under standard LD conditions and under the influence of different abiotic stress treatments (for details, see Table 1). The weight of dried leaves on the main shoot was simultaneously determined when all plants grown under the same abiotic stress condition had opened their first flowers (Table 1). The boxes delimit the 25th to the 75th percentiles of the datasets (n = 10, unless stated otherwise). The median is illustrated as a horizontal line, the mean value as a filled square, and the individual measurements as diamonds. Outlier values are crossed out. The lower and upper whiskers indicate values that differ least from the 25th percentile – 1.5 · IQR (interquartile range) or 75th percentile + 1.5 · IQR, respectively. Normal distribution of the data was tested by applying the Kolmogorov-Smirnov test. For each treatment statistical significance of the difference between homozygous *NtFT5*$^{+}$ and heterozygous *Ntft5*$^{-}$/*NtFT5*$^{+}$ plants was assessed by applying Welch's *t*-test and *P*-values were adjusted with Holm-Bonferroni correction (***P<0.001;**P<0.01).

1.1 Supplementary Tables

Table S1 | List of primers used in this study. Underlines are 5′-overhangs for cloning, which contain restriction sites (in bold). For fragment length analysis, the oligonucleotide marked with an asterisk was 5′-labeled with the fluorescent dye 6-carboxyfluorescein (6-FAM). Annealing temperatures (T) are given in °C, if the primer combinations were used for PCR.

Name	Sequence (5′ to 3′)	T [°C]	Usage
P_{NtFT5} 2.5kb for<br>P_{NtFT5} 2.5kb rev	CGCTCCTTATCATCACGTTCTTTACTCG<br>CGAAGGTCATCTCCACCAACTTCAACCCTG	68	isolation of genomic *NtFT5*<br>(P_{NtFT5})
NtFT5 5′UTR for<br>*NtFT5* intr2 rev	CTAATCGGACAGCGGGACATGATTCTTGG<br>CGATTTGACACCAATACTGCACTAGGTACG	60	isolation of genomic *NtFT5*<br>(part I)
NtFT5 intr2 for<br>*NtFT5* intr2 rev2	ATGGTTGCCTCGTGTGCTAGAATCATGC<br>AATAGTGTTGACGGAATGACCTTCAAG	53	isolation of genomic *NtFT5*<br>(part II)
NtFT5 intr2 for2<br>*NtFT5* intr3 rev	CGTACCTAGTGCAGTATTGGTGTCAAATCG<br>AATCCTGTGGTACGATGATGTTTGTGC	59.5	isolation of genomic *NtFT5*<br>(part III)
NtFT5 intr3 for<br>*NtFT5* 3′UTR rev	TCAAGGACAAAAGTGGAAATTCTCGCTC<br>AGCTGGTAATTATTAGACAAGACAGGC	61.3	isolation of genomic *NtFT5*<br>(part IV)
P_{NtFT5} for KpnI<br>P_{NtFT5} rev XhoI	AGA**GGTACC**GTCCGTACCAACTTCTTAGTTC<br>AGA**CTCGAG**GTTGATGCTTAAATAAATAAAACTAAC	50	cloning of P_{NtFT5}:*uidA*- /<br>P_{NtFT5}:*GFP_{ER}*-constructs
P_{NtFT5} diagn for<br>*uidA* screen rev	GTTGGGAAAGCAAGAGAGCG<br>TCTGCATCGGCGAACTGATCG	55	genomic identification of<br>P_{NtFT5}:*uidA* integration
M13 for<br>*GFP_{ER}* for XhoI	GTAAAACGACGGCCAG<br>AGA**CTCGAG**ATCCAAGGAGATATAAC	50	genomic identification of<br>P_{NtFT5}:*GFP_{ER}* integration
PS *NtFT5*$_{ex147..169bp}$ for<br>PS *NtFT5*$_{ex147..169bp}$ rev	ATTGCACCAACTTCAACCCTGGGC<br>AAACGCCCAGGGTTGAAGTTGGTG	-	cloning of CRISPR<br>*NtFT5* construct
NtFT1 5′UTR for<br>*NtFT1* Int1 rev	TCAAACAAAGCTATACAAACTTATCC<br>GATCCATGTAGATTACACGG	48	amplification of *NtFT1* (exon I)<br>for sequencing
NtFT2 5′UTR for<br>*NtFT2* Int1 rev	AAAGCTACACAAGCCGAC<br>CCTGGCATAAGGATCAGC	50	amplification of *NtFT2* (exon I)<br>for sequencing
NtFT3 5′UTR for<br>*NtFT3* Int1 rev	CTAAAGGTTCTGTCTATTTTTGC<br>GTCTATTATAAGCAGCCTTACC	50	amplification of *NtFT3* (exon I)<br>for sequencing
NtFT4 5′UTR for<br>*NtFT4* Int1 rev	GAAAAGTAAAGTATTTAGTGTATAG<br>ATGCGAATCAATTATAAATGG	45	amplification of *NtFT4* (exon I)<br>for sequencing
NtFT8 5′UTR for<br>*NtFT8* Int1 rev	GCACAAAAAGACATGAAGCAT<br>GAAAGATGTTATTCTTCATCGAATAG	48	amplification of *NtFT8* (exon I)<br>for sequencing
NtFT12 5′UTR for<br>*NtFT12* Int1 rev	CAATAGCTAGCTCCTCCTATAG<br>AACTTTACCGTTATTCCAAGACTC	48	amplification of *NtFT12* (exon I)<br>for sequencing
NtFT5 5′UTR for2<br>*NtFT5* intr 2 rev3	CCCTTAGACTTGTAAAAACATGC<br>ATTGTAGATAGAGTTCCTAGCG	52	amplification of *NtFT5* (exon I)<br>for sequencing
NtFT5 genomic for2<br>*NtFT5* ex I rev	*GCCATCGCCTTGTGTTAG<br>GGTATGATGTAGATTCAACCG	52	amplification of *NtFT5* (part of exon I) for fragment length analysis
Cas9 for<br>*Cas9* rev	ACGTGACCGAGGGAATGAGG<br>TTGCAGGAGATCCAGCGAGG	61	identification of genomic *Cas9*<br>integration
NtGAPDH for<br>*NtGAPDH* rev	TGGAAGAATTGGGCGATTAGTG<br>GAGCAGCAGCCTTGTCCTTG	60	genomic PCR *NtGAPDH*<br>(control)
qRT *NtFT5*/*NtomFTy* for discr<br>qRT *NtFT5*/*NtomFTy* rev discr	TCCCAAGTTATTAACCAGCCC<br>CATAGCACACAATTTCCTGGC	67	expression analysis of *NtFT5*<br>via qPCR<br>(Beinecke et al., 2018)
qRT *Nt/NtomEF-1α* for<br>qRT *Nt/NtomEF-1α* rev	AAGCTGACTGTGCTGTCCTGA<br>GGTGGTAGCATCCATCTTGTTG	66.7	expression analysis of *NtEF1α*<br>via qPCR<br>(Beinecke et al., 2018)

Table S2 | Genotypes of the seven identified *cas9*-free *NtFT5*-knockout plants (T_1 generation of L2). Summary of fragment length analysis and PCR sequencing results for the *NtFT5* gene. The relevant part of the diploid *NtFT5* locus (exon I) was amplified by PCR (primers listed in Supplementary Table S1). The analysis revealed the listed null allele variants (*Ntft5*$^{–}$) each carrying an insertion of +1 bp (A or C) and encoding a highly truncated NtFT5 protein (representatively shown for #78 (bold) in Figure 3C). None of the plants carried the wild-type allele (*NtFT5*$^{+}$) indicating the nullizygous *Ntft5*$^{–}$ genotype.

individual	*Ntft5*$^{–}$		*NtFT5*$^{+}$	genotype
#28	+1 bp (A)	+1 bp (C)	-	*Ntft5*$^{–}$
#54	+1 bp (A)	+1 bp (C)	-	*Ntft5*$^{–}$
#67	+1 bp (A)	+1 bp (C)	-	*Ntft5*$^{–}$
#77	+1 bp (A)	+1 bp (C)	-	*Ntft5*$^{–}$
**#78**	**+1 bp (A)**		**-**	***Ntft5*$^{–}$**
#79	+1 bp (A)		-	*Ntft5*$^{–}$
#94	+1 bp (A)		-	*Ntft5*$^{–}$

Table S3 | Plant genotypes of the first backcross generation (BC_1, n = 4) of backcrossed nullizygous *Ntft5*$^{–}$ T_1 plant L2 #78. Summary of fragment length analysis and PCR sequencing results for the *NtFT5* gene. The relevant part of the diploid *NtFT5* locus (exon I) was amplified for analysis by PCR (primers listed in Supplementary Table S1). Numbers indicate how many wild-type (*NtFT5*$^{+}$) alleles and null alleles (*Ntft5*$^{–}$) carrying the insertion of +1 bp (A) were detected, resulting in the listed genotypes.

individual	*Ntft5*$^{–}$	*NtFT5*$^{+}$	genotype
#1	1	1	*Ntft5*$^{–}$/*NtFT5*$^{+}$
**#2**	**1**	**1**	***Ntft5*$^{–}$/*NtFT5*$^{+}$**
#3	1	1	*Ntft5*$^{–}$/*NtFT5*$^{+}$
#4	1	1	*Ntft5*$^{–}$/*NtFT5*$^{+}$

Table S4 | Plant genotypes of the second backcross generation (BC_2, n = 150) of self-fertilized heterozygous *Ntft5⁻/NtFT5⁺* BC_1 plant #2 cultivated under LD conditions. Summary of PCR sequencing results for the *NtFT5* gene. The relevant part of the diploid *NtFT5* locus (exon I) was amplified for analysis by PCR (primers listed in Supplementary Table S1). Numbers indicate how many wild-type (*NtFT5⁺*) alleles and mutated alleles (*Ntft5⁻*) carrying the insertion of +1 bp (A) were detected, resulting in the listed genotypes.

individual	*Ntft5⁻*	*NtFT5⁺*	genotype	individual	*Ntft5⁻*	*NtFT5⁺*	genotype
#1	1	1	*Ntft5⁻/NtFT5⁺*	#38	2	-	*Ntft5⁻*
#2	-	2	*NtFT5⁺*	#39	1	1	*Ntft5⁻/NtFT5⁺*
#3	2	-	*Ntft5⁻*	#40	-	2	*NtFT5⁺*
#4	1	1	*Ntft5⁻/NtFT5⁺*	#41	2	-	*Ntft5⁻*
#5	2	-	*Ntft5⁻*	#42	1	1	*Ntft5⁻/NtFT5⁺*
#6	-	2	*NtFT5⁺*	#43	-	2	*NtFT5⁺*
#7	2	-	*Ntft5⁻*	#44	1	1	*Ntft5⁻/NtFT5⁺*
#8	2	-	*Ntft5⁻*	#45	2	-	*Ntft5⁻*
#9	1	1	*Ntft5⁻/NtFT5⁺*	#46	1	1	*Ntft5⁻/NtFT5⁺*
#10	2	-	*Ntft5⁻*	#47	-	2	*NtFT5⁺*
#11	2	-	*Ntft5⁻*	#48	1	1	*Ntft5⁻/NtFT5⁺*
#12	1	1	*Ntft5⁻/NtFT5⁺*	#49	1	1	*Ntft5⁻/NtFT5⁺*
#13	1	1	*Ntft5⁻/NtFT5⁺*	#50	1	1	*Ntft5⁻/NtFT5⁺*
#14	-	2	*NtFT5⁺*	#51	2	-	*Ntft5⁻*
#15	1	1	*Ntft5⁻/NtFT5⁺*	#52	-	2	*NtFT5⁺*
#16	1	1	*Ntft5⁻/NtFT5⁺*	#53	-	2	*NtFT5⁺*
#17	1	1	*Ntft5⁻/NtFT5⁺*	#54	1	1	*Ntft5⁻/NtFT5⁺*
#18	2	-	*Ntft5⁻*	#55	1	1	*Ntft5⁻/NtFT5⁺*
#19	-	2	*NtFT5⁺*	#56	1	1	*Ntft5⁻/NtFT5⁺*
#20	2	-	*Ntft5⁻*	#57	2	-	*Ntft5⁻*
#21	1	1	*Ntft5⁻/NtFT5⁺*	#58	1	1	*Ntft5⁻/NtFT5⁺*
#22	1	1	*Ntft5⁻/NtFT5⁺*	#59	1	1	*Ntft5⁻/NtFT5⁺*
#23	1	1	*Ntft5⁻/NtFT5⁺*	#60	-	2	*NtFT5⁺*
#24	2	-	*Ntft5⁻*	#61	1	1	*Ntft5⁻/NtFT5⁺*
#25	-	2	*NtFT5⁺*	#62	-	2	*NtFT5⁺*
#26	2	-	*Ntft5⁻*	#63	-	2	*NtFT5⁺*
#27	2	-	*Ntft5⁻*	#64	2	-	*Ntft5⁻*
#28	1	1	*Ntft5⁻/NtFT5⁺*	#65	1	1	*Ntft5⁻/NtFT5⁺*
#29	2	-	*Ntft5⁻*	#66	1	1	*Ntft5⁻/NtFT5⁺*
#30	2	-	*Ntft5⁻*	#67	2	-	*Ntft5⁻*
#31	-	2	*NtFT5⁺*	#68	2	-	*Ntft5⁻*
#32	-	2	*NtFT5⁺*	#69	-	2	*NtFT5⁺*
#33	1	1	*Ntft5⁻/NtFT5⁺*	#70	1	1	*Ntft5⁻/NtFT5⁺*
#34	2	-	*Ntft5⁻*	#71	2	-	*Ntft5⁻*
#35	-	2	*NtFT5⁺*	#72	1	1	*Ntft5⁻/NtFT5⁺*
#36	1	1	*Ntft5⁻/NtFT5⁺*	#73	-	2	*NtFT5⁺*
#37	-	2	*NtFT5⁺*	#74	-	2	*NtFT5⁺*

Table S4 | Continued.

individual	***Ntft5*** $^{-}$	***NtFT5*** $^{+}$	**genotype**	**Individual**	***Ntft5*** $^{-}$	***NtFT5*** $^{+}$	**genotype**
#75	1	1	*Ntft5* $^{-}$/*NtFT5* $^{+}$	#113	1	1	*Ntft5* $^{-}$/*NtFT5* $^{+}$
#76	2	-	*Ntft5* $^{-}$	#114	2	-	*Ntft5* $^{-}$
#77	-	2	*NtFT5* $^{+}$	#115	1	1	*Ntft5* $^{-}$/*NtFT5* $^{+}$
#78	-	2	*NtFT5* $^{+}$	#116	2	-	*Ntft5* $^{-}$
#79	2	-	*Ntft5* $^{-}$	#117	1	1	*Ntft5* $^{-}$/*NtFT5* $^{+}$
#80	-	2	*NtFT5* $^{+}$	#118	2	-	*Ntft5* $^{-}$
#81	1	1	*Ntft5* $^{-}$/*NtFT5* $^{+}$	#119	2	-	*Ntft5* $^{-}$
#82	1	1	*Ntft5* $^{-}$/*NtFT5* $^{+}$	#120	-	2	*NtFT5* $^{+}$
#83	1	1	*Ntft5* $^{-}$/*NtFT5* $^{+}$	#121	-	2	*NtFT5* $^{+}$
#84	1	1	*Ntft5* $^{-}$/*NtFT5* $^{+}$	#122	1	1	*Ntft5* $^{-}$/*NtFT5* $^{+}$
#85	1	1	*Ntft5* $^{-}$/*NtFT5* $^{+}$	#123	-	2	*NtFT5* $^{+}$
#86	1	1	*Ntft5* $^{-}$/*NtFT5* $^{+}$	#124	1	1	*Ntft5* $^{-}$/*NtFT5* $^{+}$
#87	2	-	*Ntft5* $^{-}$	#125	1	1	*Ntft5* $^{-}$/*NtFT5* $^{+}$
#88	1	1	*Ntft5* $^{-}$/*NtFT5* $^{+}$	#126	2	-	*Ntft5* $^{-}$
#89	2	-	*Ntft5* $^{-}$	#127	1	1	*Ntft5* $^{-}$/*NtFT5* $^{+}$
#90	1	1	*Ntft5* $^{-}$/*NtFT5* $^{+}$	#128	-	2	*NtFT5* $^{+}$
#91	-	2	*NtFT5* $^{+}$	#129	1	1	*Ntft5* $^{-}$/*NtFT5* $^{+}$
#92	1	1	*Ntft5* $^{-}$/*NtFT5* $^{+}$	#130	-	2	*NtFT5* $^{+}$
#93	2	-	*Ntft5* $^{-}$	#131	1	1	*Ntft5* $^{-}$/*NtFT5* $^{+}$
#94	1	1	*Ntft5* $^{-}$/*NtFT5* $^{+}$	#132	1	1	*Ntft5* $^{-}$/*NtFT5* $^{+}$
#95	2	-	*Ntft5* $^{-}$	#133	-	2	*NtFT5* $^{+}$
#96	2	-	*Ntft5* $^{-}$	#134	1	1	*Ntft5* $^{-}$/*NtFT5* $^{+}$
#97	2	-	*Ntft5* $^{-}$	#135	2	-	*Ntft5* $^{-}$
#98	2	-	*Ntft5* $^{-}$	#136	-	2	*NtFT5* $^{+}$
#99	-	2	*NtFT5* $^{+}$	#137	2	-	*Ntft5* $^{-}$
#100	1	1	*Ntft5* $^{-}$/*NtFT5* $^{+}$	#138	-	2	*NtFT5* $^{+}$
#101	2	-	*Ntft5* $^{-}$	#139	2	-	*Ntft5* $^{-}$
#102	1	1	*Ntft5* $^{-}$/*NtFT5* $^{+}$	#140	-	2	*NtFT5* $^{+}$
#103	1	1	*Ntft5* $^{-}$/*NtFT5* $^{+}$	#141	1	1	*Ntft5* $^{-}$/*NtFT5* $^{+}$
#104	-	2	*NtFT5* $^{+}$	#142	2	-	*Ntft5* $^{-}$
#105	1	1	*Ntft5* $^{-}$/*NtFT5* $^{+}$	#143	-	2	*NtFT5* $^{+}$
#106	1	1	*Ntft5* $^{-}$/*NtFT5* $^{+}$	#144	2	-	*Ntft5* $^{-}$
#107	2	-	*Ntft5* $^{-}$	#145	2	-	*Ntft5* $^{-}$
#108	-	2	*NtFT5* $^{+}$	#146	1	1	*Ntft5* $^{-}$/*NtFT5* $^{+}$
#109	1	1	*Ntft5* $^{-}$/*NtFT5* $^{+}$	#147	1	1	*Ntft5* $^{-}$/*NtFT5* $^{+}$
#110	-	2	*NtFT5* $^{+}$	#148	-	2	*NtFT5* $^{+}$
#111	1	1	*Ntft5* $^{-}$/*NtFT5* $^{+}$	#149	2	-	*Ntft5* $^{-}$
#112	1	1	*Ntft5* $^{-}$/*NtFT5* $^{+}$	#150	1	1	*Ntft5* $^{-}$/*NtFT5* $^{+}$

Table S5 | GenBank accession numbers of the *NtFT1–NtFT13* coding sequences. The sequences were used for the off-target analysis of CRISPR/Cas9-mediated genome-editing (Supplementary Figure S2).

**Gene**	**GenBank accession number**	**Reference**
NtFT1	JX679067	Harig et al., 2012
NtFT2	JX679068	Harig et al., 2012
NtFT3	JX679069	Harig et al., 2012
NtFT4	JX679070	Harig et al., 2012
NtFT5	KY306470	Beinecke et al., 2018
NtFT6	KY306472	Beinecke et al., 2018
NtFT7	KY306474	Beinecke et al., 2018
NtFT8	KY306476	Beinecke et al., 2018
NtFT9	KY306477	Beinecke et al., 2018
NtFT10	KY306478	Beinecke et al., 2018
NtFT11	KY306479	Beinecke et al., 2018
NtFT12	MH447963	Beinecke et al., 2018
NtFT13	MH447965	Beinecke et al., 2018

References

Beinecke, F. A., Grundmann, L., Wiedmann, D. R., Schmidt, F. J., Caesar, A. S., Zimmermann, M., et al. (2018). The FT/FD-dependent initiation of flowering under long-day conditions in the day-neutral species *Nicotiana tabacum* originates from the facultative short-day ancestor *Nicotiana tomentosiformis*. Plant J. 96, 329–342. doi:10.1111/tpj.14033

Harig, L., Beinecke, F. A., Oltmanns, J., Muth, J., Müller, O., Rüping, B., et al. (2012). Proteins from the FLOWERING LOCUS T-like subclade of the PEBP family act antagonistically to regulate floral initiation in tobacco. Plant J. 72, 908–21. doi:10.1111/j.1365-313X.2012.05125.x
